# Automated Classification of Homeostasis Structure in Input-Output Networks

**DOI:** 10.1007/s11538-026-01679-3

**Published:** 2026-06-11

**Authors:** Xinni Lin, Fernando Antoneli, Yangyang Wang

**Affiliations:** 1 Department of Mathematics, University of Southern California, Los Angeles, California 90007 USA; 2 Escola Paulista de Medicina, Universidade Federal de São Paulo, São Paulo, SP 04039-032 Brazil; 3 Department of Mathematics, Volen National Center for Complex Systems, Brandeis University, Waltham, Massachusetts 02453 USA

**Keywords:** Infinitesimal homeostasis, Coupled dynamical systems, Input-output network, Robust perfect adaptation

## Abstract

Homeostasis is widely observed in biological systems and refers to their ability to maintain an output quantity approximately constant despite variations in external disturbances. Mathematically, homeostasis can be formulated through an input–output function mapping an external parameter to an output variable. Infinitesimal homeostasis occurs at isolated points where the derivative of this input–output function vanishes, allowing tools from singularity theory and combinatorial matrix theory to characterize and classify homeostatic mechanisms in terms of network topology. Although the theoretical framework allows homeostasis subnetworks to be identified directly from combinatorial structures of the input–output network without numerical simulation, the required combinatorial enumeration becomes increasingly intractable as network size grows. Moreover, the reliance on advanced graph-theoretic concepts limits its broader accessibility and practical use across disciplines, particularly in biological applications. To overcome these limitations, we develop a Python-based algorithm that automates the identification of homeostasis subnetworks and their associated homeostasis conditions directly from network topology. Given an input–output network specified solely by its connectivity structure and the designation of input and output nodes, the algorithm automatically identifies the relevant graph-theoretical structures and enumerates all homeostatic mechanisms. We demonstrate the applicability of the algorithm across a range of biological examples, including small and large networks, networks with a single input parameter (with single or multiple input nodes), multiple input parameters, and cases where input and output coincide. This wide applicability stems from our extension of the theoretical framework from single-input–single-output networks to networks with multiple input nodes through an augmented single-input-node representation. The resulting computational framework provides a scalable and systematic approach to classifying homeostatic mechanisms in complex biological networks, facilitating the application of advanced mathematical theory to a broad range of biological systems.

## Introduction

Homeostasis refers to a phenomenon whereby the output *z* of a system (e.g., body temperature) maintains approximately constant on variation of an input ${\mathcal {I}}$ (e.g., environmental temperature). Mathematically, this is often represented by an *input-output function* $z({\mathcal {I}})$ that is required to be nearly constant over a range of external stimuli ${\mathcal {I}}$. Many researchers have emphasized that homeostasis is an important phenomenon in biology. For example, the extensive work of Nijhout, Reed, Best and collaborators (Nijhout et al. 2004; Reed et al. 2010; Best et al. 2009; Nijhout and Reed 2014; Nijhout et al. 2017; Reed et al. 2017; Nijhout et al. 2015, 2018) consider networks whose nodes represent the concentrations of certain biochemical substrates. Further examples include regulation of cell number and size (Lloyd 2013), the control of sleep (Wyatt et al. 1999), and the expression level regulation in housekeeping genes (Antoneli et al. 2018). The literature is huge, and these articles are a small sample.

In related work under the name of *perfect adaptation* Ma et al. (2009) identify, among all three-node enzyme networks with Michaelis-Menten chemical kinetics, two networks that are able to *adapt*, that is, to reset themselves after responding to an external stimulus. This amounts to ask for the input-output function to be independent of the external stimuli ${\mathcal {I}}$, i.e., $z=$ constant. *Perfect adaptation* has been used widely, for example, in ecology, chemistry, and control engineering (cf. Ma et al. (2009); Ang and McMillen (2013); Tang and McMillen (2016); Ferrell (2016); Qian and Vecchio (2018); Araujo and Liota (2018); Vecchio et al. (2018); Aoki et al. (2019); Hong et al. (2025); Drengstig et al. (2012)).

Golubitsky and Stewart (2017) introduced the notion of *infinitesimal homeostasis* that is intermediary between perfect adaptation and general homeostasis. Consider system of ordinary differential equations (ODEs) depending on an *input parameter* ${\mathcal {I}}$, which varies over a range of external stimuli. Suppose there is a family of equilibrium points $X({\mathcal {I}})$ and an observable $\phi$, such that the *input-output function* $z({\mathcal {I}})=\phi (X({\mathcal {I}}))$ is well-defined on the range of ${\mathcal {I}}$. In this situation, we say that the system exhibits *homeostasis* if, under variation of the input parameter ${\mathcal {I}}$, the input-output function $z({\mathcal {I}})$ remains approximately constant over the interval of external stimuli. We say that the system exhibits *infinitesimal homeostasis* at ${\mathcal {I}}_0$ if $z'({\mathcal {I}}_0) = 0$, where $\prime$ indicates differentiation with respect to ${\mathcal {I}}$. Obviously, *perfect adaptation* (or *perfect homeostasis*) occurs when $z'\equiv 0$.

Therefore, perfect homeostasis implies infinitesimal homeostasis at all points and infinitesimal homeostasis at ${\mathcal {I}}_0$ implies homeostasis in a neighborhood of ${\mathcal {I}}_0$. It is not difficult to see that the converse do not hold in both cases. One of the benefits of working with the notions of infinitesimal homeostasis is that it amounts to a very simple property to check, namely, if a derivative of a function is zero at some point. On the other hand, this simple property opens up to the possibility to apply the powerful theorems of singularity theory to describe the topological structure of input-output functions. Indeed, already in Golubitsky and Stewart (2017) the authors are able to show that scalar input-output functions $z:{\textbf{R}}\rightarrow {\textbf{R}}$ can be classified by *Elementary Catastrophe Theory* (Thom 1969, 1975; Zeeman 1977).

A particularly fruitful approach to understand the mechanisms that lead to homeostasis is to regard homeostasis as a network concept. This is motivated by the abundance of networks in biology, specially connected to ODE modeling, see e.g. Nijhout et al. (2014); Reed et al. (2017); Ferrell (2016). In fact, almost all ODE models in biology come together with a ‘wiring diagram’, or a network, describing the interactions among the elements in the model. The recently published book (Golubitsky and Stewart 2023) gives an exposition of a formal framework for studying networks of coupled ODEs, that have been developed by the authors and collaborators in the past decade. More precisely, a network of coupled ODEs is determined by directed graph whose nodes and edges are classified into types. Nodes, or cells, represent the variables of a component ODE. Edges, or arrows, between nodes represent couplings from the tail node to the head node. Nodes of the same type have the same phase spaces (up to a canonical identification); edges of the same type represent identical couplings.

In the setting of networks of coupled ODEs there is a preferable class of observables, namely the output of the node variables (coordinate functions). Network systems are distinguished from large systems by the ability to keep track of the output from each node individually. Now homeostasis can be naturally defined as the fact that the output of a node variable (the ‘output node’) is held approximately constant as other variables (other nodes) vary (perhaps wildly) under variation of an input parameter that affects another node (the ‘input node’). Placing homeostasis in the general context of network dynamics leads naturally to the methods reviewed here.

A special kind of network of coupled ODE, with two distinguished nodes (one input node and one output node) is called an *input-output network* or *single-input-single-output network*. It was introduced in (Golubitsky and Wang 2020) in their study of homeostasis on 3-node networks. Wang et al. (2021) extended the notion of *input-output network* to arbitrary large networks and developed a combinatorial theory for the classification of ‘homeostasis types’ in such networks. A *homeostasis type* is essentially a ‘combinatorial mechanism’ that causes homeostasis in the full network and is represented by specific subnetworks, called *homeostasis subnetworks*. The homeostasis subnetworks can be subdivided into two classes called *structural* and *appendage*.

The motivation for the term *structural homeostasis* comes from Reed et al. (2017), where the authors identify the feedforward loop as one of the homeostatic motifs in 3-node biochemical networks. In this case, homeostasis is caused by a ‘balance’ along the directed paths of feedforward loop, forming the ‘structural backbone’ of the input-output network. The intuition behind the term *appendage homeostasis* is that homeostasis is generated by a cycle of nodes attached to nodes in ‘structural backbone’ of the input-output network. Hence, the structural and appendage classes are abstract generalizations of the usual ‘feedforward’ and ‘feedback’ mechanisms. A striking outcome of Wang et al. (2021) is that they do not specify any homeostasis generating mechanisms at the outset and they find *a posteriori* that there are essentially only the two types of homeostasis generating mechanisms: *generalized feedback* (structural) and *generalized feedforward* (appendage).

Currently, homeostasis subnetworks are recognized by manually computing certain combinatorial structures of the input-output network (see section 2 for the definitions). However, this process would pose significant computational challenges in the case of large networks. As the network size increases, identifying these combinatorial structures becomes increasingly untractable.

***Main results of the paper***

To address this issue, in this paper we present a Python-based algorithm that automates the identification of homeostasis subnetworks of an input-output network. The main contributions of this work are as follows:

- We extend the existing theory developed for single-input-single-output network (Wang et al. 2021) to more general input-output networks with multiple inputs or multiple input nodes.
- We develop a fully automated algorithm that identifies all homeostasis subnetworks and derives the corresponding homeostasis conditions directly from network topology.
- We demonstrate the effectiveness of the approach through biologically motivated examples, including networks ranging from small to moderately large scale, with single or multiple inputs, single or multiple input nodes, as well as networks in which the input coincides with the output node.

***Structure of the paper***

The remainder of our paper is organized as follows. In Section 2.1, we give the basic definitions of infinitesimal homeostasis, input-output networks, admissible system, and the homeostasis matrix, together with a number of combinatorial terms required for the theoretical characterization of homeostasis in an input-output network. These combinatorial structures enable the identification and classification of all the homeostasis subnetwork motifs based only on the network architecture, without performing numerical simulations on model equations. In Section 2.2, we extend the theory developed for the single-input framework (Wang et al. 2021) to more general input-output networks with multiple inputs or multiple input nodes. In Section 3, we describe the implementation and usage of our Python-based classification algorithm based on the theory developed in Section 2. We illustrate the workflow using a 12-node example network whose homeostatic structure has been fully analyzed in Wang et al. (2021). In Section 4, we apply the algorithm to several biological network examples ranging from small to relatively large networks. Finally, we conclude in Section 5.

## Infinitesimal Homeostasis in Input-Output Networks

### Single-Input Single-Output Networks

We start with the case of single-input single-output input-output networks ${\mathcal {G}}$, see Golubitsky and Wang (2020); Wang et al. (2021).

The types of homeostasis that admissible systems of differential equations of ${\mathcal {G}}$ can exhibit are characterized by the topology of so called homeostasis subnetworks. Given an input-output network ${\mathcal {G}}$ there is an algorithm for determining the homoestasis subnetworks of ${\mathcal {G}}$. In order to present the algorithm, we briefly review the basic definitions and results of Wang et al. (2021).

As noted previously (Golubitsky and Stewart 2017; Reed et al. 2017; Golubitsky and Wang 2020), a straightforward application of Cramer’s rule gives a formula for determining infinitesimal homeostasis points. See Lemma 2.3.

An input-output network ${\mathcal {G}}$ is a directed graph consisting of $n+2$ nodes. There is an *input node* $\iota$, an *output node* *o*, and *n* *regulatory nodes* $\rho = (\rho _1,\ldots ,\rho _n)$. An *admissible system* associated with the input-output network ${\mathcal {G}}$ is a parameterized system of ODEs

2.1 $$\begin{aligned} \dot{X} = F(X, {\mathcal {I}}) \end{aligned}$$

where $X = (x_\iota ,x_\rho ,x_o) \in {\textbf{R}}^{n+2}$ are the node state variables, ${\mathcal {I}}\in {\textbf{R}}$ is the *external input parameter*, and $F = (f_\iota ,f_{\rho },f_o)$ is the associated vector field. Explicitly, (2.1) is the system

2.2 $$\begin{aligned} \begin{aligned} \dot{x}_\iota&= f_\iota (x_\iota ,x_\rho , x_o,{\mathcal {I}}) \\ \dot{x}_\rho&= f_\rho (x_\iota ,x_\rho ,x_o) \\ \dot{x}_o&= f_o(x_\iota ,x_\rho ,x_o) \end{aligned} \end{aligned}$$

We make the following assumptions about the vector field *F* throughout:

- The vector field *F* is smooth and has an asymptotically stable equilibrium at $(X_0,\mathcal {I}_0)$.
- $f_j$ depends on node $\ell$ only if there is an arrow in the network ${\mathcal {G}}$ from $\ell \rightarrow j$.
- $f_\iota$ is the only vector field component that depends explicitly on ${\mathcal {I}}$ and $f_{\iota ,{\mathcal {I}}}\ne 0$ generically.

We write $f_{i,x_j}$ to denote the partial derivative of $f_i$ with respect to *j* at $(X_0,{\mathcal {I}}_0)$.

#### Remark 2.1

In Wang et al. (2021) the authors explicitly exclude the possibility that the output node is one of the input nodes. This assumption is included purely for the sake of convenience. In fact, all the definitions and results remain valid (including the algorithm for classification of homeostasis subnetworks) when the input node is the same as the output nodes (see Antoneli et al. (2025) and sub-section 2.1.1). In example 4.6 we apply our algorithm and discuss a network where the input node equals the output node. $\Diamond$

It follows from the assumption that $X_0$ is a linearly stable equilibrium of (2.2) at ${\mathcal {I}}={\mathcal {I}}_0$ and the implicit function theorem that there is a unique smooth family of stable equilibria $X({\mathcal {I}})=\big (x_\iota ({\mathcal {I}}),x_\rho ({\mathcal {I}}),x_o({\mathcal {I}})\big )$ as ${\mathcal {I}}$ varies on neighborhood of ${\mathcal {I}}_0$. This allows us to define the notion of ‘infinitesimal homeostasis’ in the context of input-output networks.

#### Definition 2.2

The *input-output* function of system (2.2), at the family of equilibria $\big (X({\mathcal {I}}),{\mathcal {I}}\big )$, is the function ${\mathcal {I}}\rightarrow x_o({\mathcal {I}})$, that is, the projection of $X({\mathcal {I}})$ onto the coordinate $x_o$. We say that the input-output function $x_o({\mathcal {I}})$ exhibits *infinitesimal homeostasis* at $\mathcal {I}_0$, if

2.3 $$\begin{aligned} x_o^{\prime }(\mathcal {I}_0) = 0 \end{aligned}$$

where $\prime$ indicates differentiation with respect to ${\mathcal {I}}$. We say that the input-output function $x_o({\mathcal {I}})$ exhibits *perfect homeostasis* or *perfect adaptation* if $x_o^\prime \equiv 0$, that is, $x_o({\mathcal {I}})$ is constant. $\Diamond$

We use the following notation. Let *J* be the $(n+2)\times (n+2)$ Jacobian matrix of (2.2) and let $H$ be the $(n+1)\times (n+1)$ *homeostasis matrix* given by dropping the first row and the last column of *J*:

2.4 $$\begin{aligned} J = \left[ \begin{array}{cccccccccccc|ccc} f_{\iota , x_\iota } & f_{\iota , x_\rho } & f_{\iota , x_o} \\ f_{\rho , x_\iota } & f_{\rho , x_\rho } & f_{\rho , x_o} \\ f_{o, x_\iota } & f_{o, x_\rho } & f_{o, x_o} \end{array}\right] \quad H = \left[ \begin{array}{cccccccccccc|ccc} f_{\rho , x_\iota } & f_{\rho , x_\rho }\\ f_{o, x_\iota } & f_{o, x_\rho } \end{array}\right] \end{aligned}$$

Here all partial derivatives $f_{\ell ,x_j}$ are evaluated at the equilibrium $X_o$.

#### Lemma 2.3

Let $(X_0,{\mathcal {I}}_0)$ be an asymptotically stable equilibrium of (2.2). The input-output function $x_o({\mathcal {I}})$ satisfies

2.5 $$\begin{aligned} x_o' = \pm \frac{f_{\iota , {\mathcal {I}}}}{\det (J)} \det (H) \end{aligned}$$

Hence, ${\mathcal {I}}_0$ is a point of infinitesimal homeostasis if and only if

2.6 $$\begin{aligned} \det (H)= 0 \end{aligned}$$

at $$(X_o, {\mathcal {I}}_0)$$.

Homeostasis in a given network ${\mathcal {G}}$ can be determined by analyzing a simpler network that is obtained by eliminating certain nodes and arrows from ${\mathcal {G}}$. We call the network formed by the remaining nodes and arrows the *core subnetwork*.

#### Definition 2.4

A node $\tau$ in a network ${\mathcal {G}}$ is *downstream* from a node $\rho$ in ${\mathcal {G}}$ if there exists a path in ${\mathcal {G}}$ from $\rho$ to $\tau$. Node $\rho$ is *upstream* from node $\tau$ if $\tau$ is downstream from $\rho$. $\Diamond$

#### Definition 2.5

The input-output network ${\mathcal {G}}$ is a *core network* if every node in ${\mathcal {G}}$ is both upstream from the output node *o* and downstream from the input node $\iota$. ${\mathcal {G}}_c$ is the *core subnetwork* whose nodes are the nodes in ${\mathcal {G}}$ that are both upstream from the output and downstream from the input and whose arrows are the arrows in ${\mathcal {G}}$ whose head and tail nodes are both nodes in ${\mathcal {G}}_c$. $\Diamond$

Core networks always have that $\det (H) \ne 0$, as multivariate polynomial in $f_{\ell ,x_j}$ (see (Madeira and Antoneli, 2022, App. B)). In other words, in non-core networks $\det (H)$ might vanish identically.

The computation of infinitesimal homeostasis reduces to solving $\det (H)=0$, where *H* is the homeostasis matrix associated with the core subnetwork of ${\mathcal {G}}$. Applying Frobenius-König theory (Brualdi and Ryser 1991; Schneider 1977) to *H*, Wang et al. (2021) shows that there is a unique factorization $\det (H) = \det (B_1)\cdots \det (B_m)$. Each block submatrix $B_\eta$ is an irreducible component of *H*, with $\det (B_\eta )=0$ and $\det (B_\chi )\ne 0$ for all $\chi \ne \eta$ being a defining condition for infinitesimal homeostasis. This is generic when there is only one input parameter. Furthermore, one can associate a homeostasis subnetwork ${\mathcal {K}}_\eta$ of ${\mathcal {G}}$ with each $B_\eta$.

#### Definition 2.6

Let ${\mathcal {G}}$ be a core input-output network.

- A directed path connecting nodes $\rho$ and $\tau$ is called a *simple path* if it visits each node on the path at most once.
- An $\iota o$*-simple path* is a simple path connecting the input node $\iota$ to the output node *o*.
- A node in ${\mathcal {G}}$ is *simple* if the node lies on an $\iota o$-simple path and *appendage* if the node is not simple.
- A *super-simple* node is a simple node that lies on every $\iota o$-simple path.

Nodes $\iota$ and *o* are super-simple since by definition these nodes are on every $\iota o$-simple path. $\Diamond$

Observe that super-simple nodes are well ordered (by downstream ordering) and hence adjacent super-simple pairs of nodes can be identified.

***Appendage Homeostasis***

If $B_\eta$ corresponds to an irreducible appendage block, then the associated homeostasis subnetwork ${\mathcal {K}}_\eta$ consists of only appendage nodes and its Jacobian matrix is given by $B_\eta$. Combinatorial characterization of appendage homeostasis networks requires the following definitions.

#### Definition 2.7

Let ${\mathcal {G}}$ be a core input-output network.

- The *appendage subnetwork* ${\mathcal {A}}_{\mathcal {G}}$ of ${\mathcal {G}}$ is the subnetwork consisting of all appendage nodes and all arrows in ${\mathcal {G}}$ connecting appendage nodes.
- The *complementary subnetwork* of an $\iota o$-simple path *S* is the subnetwork ${\mathcal {C}}_S$ consisting of all nodes not on *S* and all arrows in ${\mathcal {G}}$ connecting those nodes.
- Nodes $\rho _i,\rho _j$ in ${\mathcal {A}}_{\mathcal {G}}$ are *path equivalent* if there exists paths in ${\mathcal {A}}_{\mathcal {G}}$ from $\rho _i$ to $\rho _j$ and from $\rho _j$ to $\rho _i$. An *appendage path component* is a path equivalence class in ${\mathcal {A}}_{\mathcal {G}}$.
- A *cycle* is a path whose first and last nodes are identical.
- Let ${\mathcal {K}}\subset {\mathcal {A}}_{\mathcal {G}}$ be a subnetwork. We say that ${\mathcal {K}}$ satisfies the *no cycle condition* if for every $\iota o$-simple path *S*, nodes in ${\mathcal {K}}$ do not form a cycle with nodes in ${\mathcal {C}}_S\setminus {\mathcal {K}}$. $\Diamond$

#### Remark 2.8

Nodes in the appendage subnetwork ${\mathcal {A}}_{\mathcal {G}}$ can be written uniquely as the disjoint union

2.7 $$\begin{aligned} {\mathcal {A}}_{\mathcal {G}}= ({\mathcal {A}}_1\dot{\cup }\cdots \dot{\cup }{\mathcal {A}}_s)\; \dot{\cup }\;({\mathcal {B}}_1\dot{\cup }\cdots \dot{\cup }{\mathcal {B}}_t) \end{aligned}$$

where each ${\mathcal {A}}_i$ is an appendage path component that satisfies the no cycle condition and each ${\mathcal {B}}_i$ is an appendage path component that violates the no cycle condition. Moreover, each ${\mathcal {A}}_i$ (resp. ${\mathcal {B}}_i$) can be viewed as a subnetwork of ${\mathcal {A}}_{\mathcal {G}}$ by including the arrows in ${\mathcal {A}}_{\mathcal {G}}$ that connect nodes in ${\mathcal {A}}_i$ (resp. ${\mathcal {B}}_i$). We call ${\mathcal {A}}_i$ a *no cycle appendage path component* and ${\mathcal {B}}_i$ a *cycle appendage path component*. $\Diamond$

***Structural Homeostasis***

If $B_\eta$ corresponds to an irreducible structural block, then ${\mathcal {K}}_\eta$ has two adjacent super-simple nodes and these super-simple nodes are the input node $\ell$ and the output node *j* in ${\mathcal {K}}_\eta$. In addition, the network ${\mathcal {K}}_\eta$ contains no backward arrows. That is, no arrows of ${\mathcal {K}}_\eta$ go into the input node $\ell$ nor out of the output node *j*.

#### Definition 2.9

The *structural subnetwork* ${\mathcal {S}}_{\mathcal {G}}$ of ${\mathcal {G}}$ is the subnetwork whose nodes are either simple or in a cycle appendage path component ${\mathcal {B}}_i$ (see Remark 2.8) and whose arrows are arrows in ${\mathcal {G}}$ that connect nodes in ${\mathcal {S}}_{\mathcal {G}}$. $\Diamond$

All structural homeostasis subnetworks are contained in ${\mathcal {S}}_{\mathcal {G}}$, which is an input-output network. That is, ${\mathcal {G}}$ and ${\mathcal {S}}_{\mathcal {G}}$ have the same simple, super-simple, input, and output nodes. Moreover, every non-super-simple simple node lies between two adjacent super-simple nodes.

#### Definition 2.10

Let $\rho _1, \rho _2$ be adjacent super-simple nodes.

- A simple node $\rho$ is *between* $\rho _1$ and $\rho _2$ if there exists an $\iota o$-simple path that includes $\rho _1$ to $\rho$ to $\rho _2$ in that order.
- The *super-simple subnetwork*, denoted ${\mathcal {L}}(\rho _1,\rho _2)$, is the subnetwork whose nodes are simple nodes between $\rho _1$ and $\rho _2$ and whose arrows are arrows of ${\mathcal {G}}$ connecting nodes in ${\mathcal {L}}(\rho _1,\rho _2)$. $\Diamond$

It follows that all ${\mathcal {L}}(\rho _1,\rho _2)$ are contained in ${\mathcal {S}}_{\mathcal {G}}$ and each appendage node in ${\mathcal {S}}_{\mathcal {G}}$ connects to exactly one ${\mathcal {L}}$. Hence, a super-simple subnetwork ${\mathcal {L}}\subset {\mathcal {S}}_{\mathcal {G}}$ can be expanded to a super-simple structural subnetwork ${\mathcal {L}}'\subset {\mathcal {S}}_{\mathcal {G}}$ as follows.

#### Definition 2.11

Let $\rho _1$ and $\rho _2$ be adjacent super-simple nodes in ${\mathcal {G}}$. The *super-simple structural subnetwork* ${\mathcal {L}}'(\rho _1,\rho _2)$ is the input-output subnetwork consisting of nodes in ${\mathcal {L}}(\rho _1,\rho _2)\cup {\mathcal {B}}$ where ${\mathcal {B}}$ consists of all appendage nodes that form cycles with nodes in ${\mathcal {L}}(\rho _1,\rho _2)$; that is, all cycle appendage path components that connect to ${\mathcal {L}}(\rho _1,\rho _2)$. Arrows of ${\mathcal {L}}'(\rho _1,\rho _2)$ are arrows of ${\mathcal {G}}$ that connect nodes in ${\mathcal {L}}'(\rho _1,\rho _2)$. Thus, $\rho _1$ is the input node and $\rho _2$ is the output node of ${\mathcal {L}}'(\rho _1,\rho _2)$. $\Diamond$

#### Networks with Input = Output

Andrade et al. (2022); Antoneli et al. (2025, 2026a) provide a detailed discussion of the infinitesimal homeostasis formalism in the case where the input and the output nodes are the same. We briefly recall the main points in the following.

When the network $\mathcal {G}$ has the same node as the input and output nodes the corresponding variables coincide $x_\iota =x_o$ and we call the network an *input=output network* and $\iota$ the input $=$ output node.

In this case the vector of state variable is $X=(x_{\iota },x_{\rho })\in \mathbb {R}\times \mathbb {R}^N$ and the system of ODE’s (2.1) becomes

2.8 $$\begin{aligned} \begin{aligned} \dot{x}_{\iota }&= f_{\iota }(x_{\iota }, x_{\rho }, \mathcal {I}) \\ \dot{x}_{\rho }&= f_{\rho }(x_{\iota }, x_{\rho })\\ \end{aligned} \end{aligned}$$

Let *J* be the $(N+1)\times (N+1)$ Jacobian matrix of an admissible vector field $F=(f_{\iota },f_{\sigma })$, that is,

2.9 $$\begin{aligned} J = \begin{pmatrix} f_{\iota , x_{\iota }} & f_{\iota , x_\rho } \\ f_{\rho , x_{\iota }} & f_{\rho , x_\rho } \end{pmatrix} \end{aligned}$$

Now, the $N\times N$ *homeostasis matrix* *H* is obtained from *J* by removing the *first row* and the *first column*:

2.10 $$\begin{aligned} H = \begin{pmatrix} f_{\rho , x_\rho } \end{pmatrix} \end{aligned}$$

In both (2.9) and (2.10) partial derivatives $f_{\ell ,x_j}$ are evaluated at the equilibrium $\big (X(\mathcal {I}),\mathcal {I}\big )$.

The main difference between the homeostasis matrix (2.10) and the homeostasis matrix of network with distinct input and output nodes is that the former contains only the partial derivatives associated with the regulatory nodes, while the latter contains partial derivatives involving the input and the output nodes, as well. In fact, the matrix *H* in eq. (2.10) is the Jacobian matrix of the subnetwork generated by the regulatory nodes.

As we have seen, the classification of homeostasis subnetworks stats with the reduction the the core subnetwork. The same definition of core network and core-equivalence apply to an input $=$ output network. However, in the input $=$ output case, the condition that a node $\rho$ that is both upstream from the output node and downstream from the input node takes a special form.

##### Lemma 2.12

Let $\mathcal {G}$ be an input $=$ output network, with input $=$ output node $\iota$. A regulatory node $\rho$ belongs to the core subnetwork $\mathcal {G}_c$ if and only if $\rho$ belongs to a cycle that contains the input $=$ output node $\iota$.

##### Proof

A directed path starting and ending at the same node is a cycle. $\square$

##### Corollary 2.13

An input $=$ output network $\mathcal {G}$ is a core network if and only if every regulatory node of $\mathcal {G}$ belongs to a cycle that contains the input $=$ output node.

In a core network $\mathcal {G}$ where the input node is distinct from the output node, a simple node is always downstream from the input node and upstream from the output node, but not ‘the other way around’. That is, if a node is *downstream* the *output node* and/or *upstream* the *input node* then it must be an *appendage node*. Indeed, if $\mathcal {G}$ is a core network then every node is downstream from the input node and upstream from the output node. Then a node that satisfies ‘the other way around’ condition above must be on an $\iota o$-path that cycles around the input and/or the output node. We note the not every appendage node is of this type, that is, it may be on an $\iota o$-path that cycles around other regulatory nodes. Hence, we have the following.

##### Lemma 2.14

Let $\mathcal {G}$ be a core input $=$ output network. Then every node of $\mathcal {G}$ is appendage.

##### Proof

Since every node in a core input $=$ output network forms a cycle with the input $=$ output node it follows that every node is *downstream* the *output node* and *upstream* the *input node*. Thus, every node is appendage. $\square$

##### Corollary 2.15

Structural homeostasis does not exist in input $=$ output networks.

The next theorem summarizes the classification of homeostasis subnetworks of an input $=$ output network.

##### Theorem 2.16

If $\mathcal {G}$ is an input $=$ output network then the appendage subnetwork of $\mathcal {G}$ is exactly the subnetwork of $\mathcal {G}$ generated by all regulatory nodes. Moreover, the irreducible factors of $\det (H)$ correspond to the appendage path components $\mathcal {A}_k$ and are given by $\det (J({\mathcal {A}_k}))$. Here $J({\mathcal {A}_k})$ is the jacobian matrix associated with the network $\mathcal {A}_k$.

##### Proof

By lemma 2.14 it follows that the set of nodes of $\mathcal {A}_\mathcal {G}$ is exactly the set of regulatory nodes. Hence they generate the same network. The, it follows from (Wang et al., 2021, Thm. 7.1), that all irreducible blocks of the homeostasis matrix correspond to the appendage path components. Moreover, since there are no simple nodes the “no cycle condition” is trivially satisfied by all appendage path components. Finally, it follows from (Wang et al., 2021, Thm. 5.4) that the corresponding irreducible factor of $\det (H)$ is of the form $\det (J({\mathcal {A}_k}))$. $\square$

#### Algorithm for the Classification of Homeostasis Subnetworks

Here is the algorithm for enumerating subnetworks corresponding to the *m* homeostasis blocks (see Wang et al. (2021)).

***Step 1:***

Determining the appendage homeostasis subnetworks from ${\mathcal {A}}_{\mathcal {G}}$. Let

2.11 $$\begin{aligned} {\mathcal {A}}_1\;,\ldots ,\;{\mathcal {A}}_s \end{aligned}$$

be the no cycle appendage path components of ${\mathcal {A}}_{\mathcal {G}}$ (see Remark 2.8). These appendage path components are the subnetworks ${\mathcal {K}}_\eta$ that correspond to appendage homeostasis blocks. In addition, there are *s* independent defining conditions for appendage homeostasis given by the determinants of the Jacobian matrices $\det (J_{{\mathcal {A}}_i}) = 0$ for $i = 1,\ldots , s$.

***Step 2:***

Determining the structural homeostasis subnetworks from ${\mathcal {S}}_{\mathcal {G}}$. Let $\iota = \rho _1> \rho _2> \ldots > \rho _{q+1} = o$ be the super-simple nodes in ${\mathcal {S}}_{\mathcal {G}}$ in downstream order. Then, up to core equivalence, the *q* super-simple structural subnetworks

2.12 $$\begin{aligned} {\mathcal {L}}'(\iota ,\rho _2),\; {\mathcal {L}}'(\rho _2,\rho _3) \;,\ldots ,\; {\mathcal {L}}'(\rho _{q-1},\rho _q),\; {\mathcal {L}}'(\rho _q,o) \end{aligned}$$

are the subnetworks ${\mathcal {K}}_\eta$ that correspond to structural homeostasis blocks. In addition, there are *q* defining conditions for structural homeostasis blocks given by the determinants of the homeostasis matrices of the input-output networks: $\det \big (H({\mathcal {L}}'(\rho _i,\rho _{i+1}))\big ) = 0$ for $i = 1,\ldots , q$.

Therefore, the $m = s + q$ subnetworks listed in (2.11) and (2.12) enumerate the appendage and structural homeostasis subnetworks in ${\mathcal {G}}$.

### Generalization to Multiple-Input Single-Output Networks

Here we explain how to extend the theory developed for single-input framework (algorithm 2.1.2) to more general input-output networks, by reducing to the single-input single-output case.

The first step is to consider single-input single-output input-output network with multiple input nodes, but a single input parameter. Then we can deal with the general case of multiple-input single-output input-output networks, that is, input-output network with multiple input nodes and multiple input parameters.

#### Single-Input Single-Output Networks with Multiple Input Nodes

In Madeira and Antoneli (2022), the authors extend the approach of Wang et al. (2021) to deal with the multiple input node case. They consider the influence of the input parameter on each input node separately by constructing a distinguished set of subnetworks with a single input node. This strategy effectively reduces the problem to the single input node case. Then they show how to put together the classification of homeostasis subnteworks of the the networks in the distinguished set of subnetworks with a single input node.

Here, we will present an alternative approach to the classification of the homeostasis subnetworks of networks with multiple input nodes that allows us to directly apply algorithm 2.1.2. Let us briefly recall some definitions and results from Madeira and Antoneli (2022) before introducing the alternative approach.

A *multiple input-node input-output network* is a network $\mathcal {G}$ with *n* distinguished *input nodes* $\iota =\{\iota _{1}, \ldots , \iota _{n}\}$, all of them associated to the same input parameter $\mathcal {I}$, one distinguished *output node* *o*, and *N* *regulatory nodes* $\rho =\{\rho _1,\ldots ,\rho _N\}$. The associated network systems of differential equations have the form

2.13 $$\begin{aligned} \begin{aligned} \dot{x}_{\iota }&= f_{\iota }(x_{\iota }, x_{\rho }, x_{o}, \mathcal {I}) \\ \dot{x}_{\rho }&= f_{\rho }(x_{\iota }, x_{\rho }, x_{o})\\ \dot{x}_{o}&= f_{o}(x_{\iota }, x_{\rho }, x_{o}) \end{aligned} \end{aligned}$$

where $\mathcal {I}\in \mathbb {R}$ is an *external input parameter* and $X=(x_{\iota },x_{\rho },x_o)\in \mathbb {R}^n\times \mathbb {R}^N\times \mathbb {R}$ is the vector of state variables associated to the network nodes. Finally, assume that ${\mathcal {G}}$ satisfies the following condition: the output node is downstream from all input nodes.

We write a vector field associated with the system (2.13) as

$$ F(X,\mathcal {I})=(f_{\iota }(X,\mathcal {I}),f_\rho (X),f_o(X)) $$

and call it an *admissible vector field* for the network $\mathcal {G}$.

Let $f_{j,x_\ell }$ denote the partial derivative of the $j^{th}$ node function $f_j$ with respect to the $\ell ^{th}$ node variable $x_\ell$. We make the following assumptions about the vector field *F* throughout:

- The vector field *F* is smooth and has an asymptotically stable equilibrium at $(X^*,\mathcal {I}^*)$. Therefore, by the implicit function theorem, there is a function $\tilde{X}(\mathcal {I})$ defined in a neighborhood of $\mathcal {I}^*$ such that $\tilde{X}(\mathcal {I}^*) = X^*$ and $F(\tilde{X}(\mathcal {I}), \mathcal {I}) \equiv 0$.
- The partial derivative $f_{j,x_\ell }$ can be non-zero only if the network $\mathcal {G}$ has an arrow $\ell \rightarrow j$, otherwise $f_{j,x_\ell } \equiv 0$.
- Only the input node coordinate functions $f_{\iota _m}$ depend on the external input parameter $\mathcal {I}$ and the partial derivative of $f_{\iota _m,\mathcal {I}}$ generically satisfies
  2.14 $$\begin{aligned} f_{\iota _m,\mathcal {I}} \ne 0. \end{aligned}$$

Let *J* be the $(n+N+1)\times (n+N+1)$ Jacobian matrix of an admissible vector field $F=(f_{\iota },f_{\sigma },f_{o})$, that is,

2.15 $$\begin{aligned} J = \begin{bmatrix} f_{\iota , x_\iota } & f_{\iota , x_\rho } & f_{\iota , x_o} \\ f_{\rho , x_\iota } & f_{\rho , x_\rho } & f_{\rho , x_o} \\ f_{o, x_\iota } & f_{o, x_\rho } & f_{o, x_o} \end{bmatrix} \end{aligned}$$

The $(n+N+1)\times (n+N+1)$ matrix $\langle H \rangle$ obtained from *J* by replacing the last column by $(-f_{\iota ,\mathcal {I}},0,0)^t$, is called *generalized homeostasis matrix*:

2.16 $$\begin{aligned} \langle H \rangle = \begin{bmatrix} f_{\iota , x_\iota } & f_{\iota , x_\rho } & -f_{\iota , \mathcal {I}} \\ f_{\rho , x_\iota }& f_{\rho , x_\rho } & 0 \\ f_{o, x_\iota } & f_{o, x_\rho } & 0 \end{bmatrix} = \begin{bmatrix} f_{\iota _{1}, x_{\iota _{1}}} & \cdots & f_{\iota _{1}, x_{\iota _{n}}} & f_{\iota _{1}, x_{\rho }} & - f_{\iota _{1}, \mathcal {I}} \\ \vdots & \ddots & \vdots & \vdots & \vdots \\ f_{\iota _{n}, x_{\iota _{1}}} & \cdots & f_{\iota _{n}, x_{\iota _{n}}} & f_{\iota _{n}, x_{\rho }} & - f_{\iota _{n}, \mathcal {I}} \\ f_{\rho , x_{\iota _{1}}} & \cdots & f_{\rho , x_{\iota _{n}}} & f_{\rho , x_{\rho }} & 0 \\ f_{o, x_{\iota _{1}}} & \cdots & f_{o, x_{\iota _{n}}} & f_{o, x_{\rho }} & 0 \end{bmatrix} \end{aligned}$$

Here all partial derivatives $f_{\ell ,x_j}$ are evaluated at $\big (\tilde{X}(\mathcal {I}),\mathcal {I}\big )$.

##### Lemma 2.17

The input-output function $x_o(\mathcal {I})$ satisfies

2.17 $$\begin{aligned} x_o'(\mathcal {I}) = \frac{\det \!\big (\langle H \rangle \big ) }{\det (J)} \end{aligned}$$

Here $\det (J)$ and $\det \!\big (\langle H \rangle \big )$ are evaluated at $\big (\tilde{X}(\mathcal {I}),\mathcal {I}\big )$. Hence, $\mathcal {I}_0$ is a point of infinitesimal homeostasis if and only if

2.18 $$\begin{aligned} \det \!\big (\langle H \rangle \big ) = 0 \end{aligned}$$

at the equilibrium $\big (\tilde{X}(\mathcal {I}_0),\mathcal {I}_0\big )$.

By expanding $\det (\langle H \rangle )$ with respect to the last column and each $\iota _k$ (input) row one obtains

2.19 $$\begin{aligned} \det \!\big (\langle H \rangle \big ) = \sum _{m=1}^n \pm f_{\iota _m,\mathcal {I}} \det (H_{\iota _m}) \end{aligned}$$

The *partial homeostasis matrix* $H_{\iota _m}$ is obtained from the Jacobian matrix *J* of *F* by dropping the last column and the $\iota _m$ row; see (2.20) below.

2.20 $$\begin{aligned} H_{\iota _{m}} = \begin{bmatrix} f_{\iota _{1}, x_{\iota _{1}}} & \cdots & f_{\iota _{1}, x_{\iota _{n}}} & f_{\iota _{1}, x_{\rho }}\\ \vdots & \ddots & \vdots & \vdots \\ f_{\iota _{m-1}, x_{\iota _{1}}} & \cdots & f_{\iota _{m-1}, x_{\iota _{n}}} & f_{\iota _{m-1}, x_{\rho }} \\ f_{\iota _{m+1}, x_{\iota _{1}}} & \cdots & f_{\iota _{m+1}, x_{\iota _{n}}} & f_{\iota _{m+1}, x_{\rho }} \\ \vdots & \ddots & \vdots & \vdots \\ f_{\iota _{n}, x_{\iota _{1}}} & \cdots & f_{\iota _{n}, x_{\iota _{n}}} & f_{\iota _{n}, x_{\rho }} \\ f_{\rho , x_{\iota _{1}}} & \cdots & f_{\rho , x_{\iota _{n}}} & f_{\rho , x_{\rho }} \\ f_{o, x_{\iota _{1}}} & \cdots & f_{o, x_{\iota _{n}}} & f_{o, x_{\rho }} \end{bmatrix} \end{aligned}$$

Note that when there is a single input node, i.e. $n=1$, Lemma 2.17 gives the corresponding result obtained in Wang et al. (2021). In this case, there is only one matrix $H_{\iota _m}=H$, which reduces to the *homeostasis matrix* from single input node case.

The generalization of the notion of core network to the multiple input nodes is straightforward.

##### Definition 2.18

Let $\mathcal {G}$ be a network with input nodes $\iota _{1}, \ldots , \iota _{n}$ and output node *o*. We call $\mathcal {G}$ a *core network* if every node in $\mathcal {G}$ is upstream from *o* and downstream from at least one input node. ${\mathcal {G}}_c$ is the *core subnetwork* whose nodes are the nodes in ${\mathcal {G}}$ that are both upstream from *o* and downstream from at least one input node and whose arrows are the arrows in ${\mathcal {G}}$ whose head and tail nodes are both nodes in ${\mathcal {G}}_c$. $\Diamond$

In Madeira and Antoneli (2022) the authors proceed to extend all the combinatorial properties of the single input node case to the multiple input node case, culminating with the definitions of homeostasis subnetworks. Then the classification proceeds by carefully analyzing how these notions relate with the corresponding notions on each single input node subnetwork of the distinguished set.

Now, we are in position to describe our new approach to the classification of the homeostasis subnetwork of networks with multiple input nodes (see Theorem 2.19 below).

Specifically, we augment the network ${\mathcal {G}}$ by introducing a new input node $x_{I}$ that connects to all original input nodes and whose dynamics enforces $x_{I}(t) = \mathcal {I}$ asymptotically. This construction effectively converts an $n+N$-node multiple input node network to an $n+N+1$-node single input node network, allowing the theory and classification results we developed in Wang et al. (2021) for single input node networks to be applied directly.

##### Theorem 2.19

Let $\mathcal {G}$ be a single-input input-output network with *n* distinguished input nodes $\iota = (\iota _1, \dots , \iota _n)$ receiving the same input from $\mathcal {I}\in \mathbb {R}$, *N* regulatory nodes $\rho$, and one distinguished output node *o*. Construct the *augmented network* $\mathcal {G}^\diamond$ by introducing a new input node *I* and converting the original input nodes $\iota = (\iota _1, \dots , \iota _n)$ into regulatory nodes, by adding directed edges $I \rightarrow \iota _j$ for $j = 1,\dots ,n$. Extend the vector field $F=(f_{\iota },f_{\rho },f_o)$ to $F^\diamond =(f_{I},f_{\iota },f_{\rho },f_o)$ in such a way that the equation for the new input node variable $\dot{x}_I(t)=f_I(x_{I},\mathcal {I})$ enforces $x_I(t)\equiv {\mathcal {I}}$ asymptotically. Then the infinitesimal homeostasis matrix $H^\diamond$ of $\mathcal {G}^\diamond$ is identical to the generalized homeostasis matrix $\langle H \rangle$ of $\mathcal {G}$ given by (2.16), up to a sign difference.

##### Proof

Let $H^\diamond$ be the homeostasis matrix for the augmented network $\mathcal {G}^\diamond$. We prove below that $\det (H^\diamond ) = \det (\langle H \rangle )$, as polynomials in $(f_{\ell ,x_j})$, up to a sign. Consequently, both formulations yield identical infinitesimal homeostasis conditions. We define the dynamics of the new input node as

$$ \dot{x}_{I}=f_I(x_{I},\mathcal {I}) := \mathcal {I}-x_I, $$

which ensures $x_I(t) = \mathcal {I}$ asymptotically. Moreover, because of the skew product form of $F^\diamond$, if the original system *F* is stable at an equilibrium $(X_0,{\mathcal {I}}_0)$ then the extended system $F^\diamond$ is stable at the corresponding equilibrium $({\mathcal {I}}_0,X_0,{\mathcal {I}}_0)$. Other choices of dynamics are possible. This is one working example. We then rewrite dynamics for the original set of input nodes as

$$ \dot{x}_{\iota } = f_{\iota }(x_\iota ,x_\rho ,x_o, x_I), $$

which now become regulatory nodes that do not directly depend on the input parameter $\mathcal {I}$. The Jacobian matrix $J^\diamond$ of $\mathcal {G}^\diamond$ becomes:

$$ J^\diamond = \begin{bmatrix} f_{I, x_{I}} & f_{I, x_{\iota }} & f_{I, x_{\rho }} & f_{I, x_o} \\ f_{\iota , x_{I}} & f_{\iota , x_{\iota }} & f_{\iota , x_{\rho }} & f_{\iota , x_o} \\ f_{\rho , x_{I}} & f_{\rho , x_{\iota }} & f_{\rho , x_{\rho }} & f_{\rho , x_o} \\ f_{o, x_{I}} & f_{o, x_{\iota }} & f_{o, x_{\rho }} & f_{o, x_o} \end{bmatrix} \quad =\quad \begin{bmatrix} f_{I, x_{I}} & 0 & 0 & 0 \\ f_{\iota , x_{I}} & f_{\iota , x_{\iota }} & f_{\iota , x_{\rho }} & f_{\iota , x_o} \\ 0 & f_{\rho , x_{\iota }} & f_{\rho , x_{\rho }} & f_{\rho , x_o} \\ 0 & f_{o, x_{\iota }} & f_{o, x_{\rho }} & f_{o, x_o} \end{bmatrix} $$

By Lemma 2.3, the input-output function $x_o(\mathcal {I})$ of this augmented network satisfies

$$ x_o'=\pm \frac{f_{I,\mathcal {I}}}{\det (J^\diamond )}\det (H^\diamond ) = \pm \frac{\det (H^\diamond )}{\det (J)} $$

where $H^\diamond$ is obtained from removing the first row and the last column of $J^\diamond$:

2.21 $$\begin{aligned} \begin{aligned} H^\diamond&= \begin{bmatrix} f_{\iota , x_{I}} & f_{\iota , x_{\iota }} & f_{\iota , x_{\rho }}\\ 0 & f_{\rho , x_{\iota }} & f_{\rho , x_{\rho }}\\ 0 & f_{o, x_{\iota }} & f_{o, x_{\rho }} \end{bmatrix} \quad = \quad \begin{bmatrix} f_{\iota , \mathcal {I}} & f_{\iota , x_{\iota }} & f_{\iota , x_{\rho }}\\ 0 & f_{\rho , x_{\iota }} & f_{\rho , x_{\rho }}\\ 0 & f_{o, x_{\iota }} & f_{o, x_{\rho }} \end{bmatrix} \end{aligned} \end{aligned}$$

The second equality follows that, at the stable equilibrium, $x_I=\mathcal {I}$. Through two column exchanges, we obtain that $\det (H^\diamond ) = \det (\langle H \rangle )$. $\square$

Since the multivariate polynomial $\det (H^\diamond )$ have the same irreducible factors of $\det (\langle H \rangle )$, we can apply algorithm 2.1.2 to the augmented network ${\mathcal {G}}^\diamond$. Moreover, it follows from (Madeira and Antoneli, 2022, Thm. 3.26) that there exist exactly one irreducible factor of $\det (\langle H \rangle )$ that contains all terms of the form $f_{\iota _i,{\mathcal {I}}}$. This irreducible factor is associated with the *input counterweight* homeostasis type. In the irreducible decomposition of $\det (H^\diamond )$ the input counterweight homeostasis type corresponds to one of the structural homeostasis types, the one associated with the unique irreducible factor that contains all terms of the form $f_{\iota _i,x_I}$ (since these terms correspond to $f_{\iota _i,{\mathcal {I}}}$).

It may happen that the output node coincides with one of the input nodes $\iota _j$. Then, in principle, one would have to take into account the observations from sub-section 2.1.1. However, it is easy to see that the augmented network always has the new input node distinct from the output node and hence one is back to the usual case where the input and the output nodes are distinct (Wang et al. 2021).

#### Multiple-Input Single-Output Networks

In Madeira and Antoneli (2024), the authors extend the approach of Wang et al. (2021); Madeira and Antoneli (2022) to deal with the general multiple-input single output case. Here, we will show that algorithm 2.1.2 can be applied multiple times to certain single-input single-output networks and hence obtain a classification of homeostasis types in this case, as well.

Let us briefly recall some definitions and results from Madeira and Antoneli (2024) before explaining the strategy to apply algorithm 2.1.2 to obtain a classification of homeostasis types.

A *multiple-input single-output* network, or simply a *multiple inputs network*, is a network $\mathcal {G}$ with *n* distinguished *input nodes* $\iota =\{\iota _{1}, \iota _{2}, \ldots , \iota _{n}\}$, all of them associated to at least one input parameter $\mathcal {I}_{M}$, $M = 1, \ldots , N$, one distinguished *output node* *o*, and *r* *regulatory nodes* $\rho =\{\rho _1,\ldots ,\rho _r\}$. The associated system of differential equations has the form

2.22 $$\begin{aligned} \begin{aligned} \dot{x}_{\iota }&= f_{\iota }(x_{\iota }, x_{\rho }, x_{o}, {\boldsymbol{\mathcal {I}}}) \\ \dot{x}_{\rho }&= f_{\rho }(x_{\iota }, x_{\rho }, x_{o})\\ \dot{x}_{o}&= f_{o}(x_{\iota }, x_{\rho }, x_{o}) \end{aligned} \end{aligned}$$

where ${\boldsymbol{\mathcal {I}}} = (\mathcal {I}_{1}, \cdots , \mathcal {I}_{N}) \in \mathbb {R}^{N}$ is the vector of *input parameters*, or simple the vector of *inputs*, and $X=(x_{\iota },x_{\rho },x_o)\in \mathbb {R}^n\times \mathbb {R}^r \times \mathbb {R}$ is the vector of state variables associated to the network nodes.

We write a vector field associated with the system (2.22) as

$$ F(X,{\boldsymbol{\mathcal {I}}})=(f_{\iota }(X,{\boldsymbol{\mathcal {I}}}),f_\rho (X),f_o(X)) $$

and call it an *admissible vector field* for the network $\mathcal {G}$.

Let $f_{j,x_i}$ denote the partial derivative of the $j^{th}$ node function $f_j$ with respect to the $i^{th}$ node variable $x_i$. We make the following assumptions about the vector field *F* throughout:

- The vector field *F* is smooth and has a linearly stable equilibrium at $(X^*,{\boldsymbol{\mathcal {I}}}^*)$. Therefore, by the implicit function theorem, there is a function $\tilde{X}({\boldsymbol{\mathcal {I}}})$ defined in a neighborhood of ${\boldsymbol{\mathcal {I}}}^*$ such that $\tilde{X}({\boldsymbol{\mathcal {I}}}^*) = X^*$ and $F(\tilde{X}({\boldsymbol{\mathcal {I}}}), {\boldsymbol{\mathcal {I}}}) \equiv 0$.
- The partial derivative $f_{j,x_i}$ can be non-zero only if the network $\mathcal {G}$ has an arrow $i\rightarrow j$, otherwise $f_{j,x_i} \equiv 0$.
- Only the input node coordinate functions $f_{\iota _k}$ depend on at least one of the components of the vector of input parameters ${\boldsymbol{\mathcal {I}}}$ and the partial derivative of $f_{\iota _k,\mathcal {I}_{M}}$ generically satisfies
  2.23 $$\begin{aligned} \frac{\partial f_{\iota _{k}}}{\partial \mathcal {I}_{M}} = f_{\iota _k,\mathcal {I}_{M}} \ne 0. \end{aligned}$$
  for some $$M = 1, \ldots , N$$.

Now the input-output function $x_o$ is multivariate, that is $x_o:\mathbb {R}^N\rightarrow \mathbb {R}$. Infinitesimal homeostasis in a multiple inputs network is given by the critical points of $x_o(\mathcal {I})$, namely, the zeros of the gradient vector

2.24 $$\begin{aligned} \nabla x_{o} = \left( \frac{\partial x_{o}}{\partial \mathcal {I}_{1}}, \frac{\partial x_{o}}{\partial \mathcal {I}_{2}}, \cdots , \frac{\partial x_{o}}{\partial \mathcal {I}_{N}} \right) \end{aligned}$$

Let *J* be the $(n+r+1)\times (n+r+1)$ Jacobian matrix of an admissible vector field $F=(f_{\iota },f_{\sigma },f_{o})$, that is,

2.25 $$\begin{aligned} J = \begin{pmatrix} f_{\iota , x_\iota } & f_{\iota , x_\rho } & f_{\iota , x_o} \\ f_{\rho , x_\iota } & f_{\rho , x_\rho } & f_{\rho , x_o} \\ f_{o, x_\iota } & f_{o, x_\rho } & f_{o, x_o} \end{pmatrix} \end{aligned}$$

For each $1 \le M \le N$, consider the $(n+r+1)\times (n+r+1)$ matrix $\langle H_M \rangle$ obtained from *J* by replacing the last column by $(-f_{\iota ,\mathcal {I}_M},0,0)^t$, which is called $\mathcal {I}_M$*-generalized homeostasis matrix*:

2.26 $$\begin{aligned} \langle H_M \rangle = \begin{pmatrix} f_{\iota , x_\iota } & f_{\iota , x_\rho } & -f_{\iota , \mathcal {I}_M} \\ f_{\rho , x_\iota }& f_{\rho , x_\rho } & 0 \\ f_{o, x_\iota } & f_{o, x_\rho } & 0 \end{pmatrix} \end{aligned}$$

Here all partial derivatives $f_{\ell ,x_j}$ are evaluated at $\big (\tilde{X}({\boldsymbol{\mathcal {I}}}),{\boldsymbol{\mathcal {I}}}\big )$.

##### Lemma 2.20

Let $x_o({\boldsymbol{\mathcal {I}}})$ be input-output function of a multiple inputs network. The partial derivative of $x_o({\boldsymbol{{\mathcal {I}}}})$ with respect to the *M*-th parameter $\mathcal {I}_M$ satisfies

2.27 $$\begin{aligned} \frac{\partial x_o\;}{\partial \mathcal {I}_M} = \frac{\det \langle H_M \rangle }{\det (J)} \end{aligned}$$

Here $\det (J)$ and $\det \langle H_M \rangle$ are evaluated at the equilibrium point $\tilde{X}({\boldsymbol{{\mathcal {I}}}})$. Hence,

2.28 $$\begin{aligned} \nabla x_{o} = \frac{1}{\det (J)}\left( \det \langle H_{1} \rangle , \det \langle H_{2} \rangle , \ldots , \det \langle H_{N} \rangle \right) \end{aligned}$$

Moreover, ${\boldsymbol{{\mathcal {I}}}}^0$ is a point of infinitesimal homeostasis if and only if

2.29 $$\begin{aligned} \det \langle H_M \rangle = 0 \qquad {\text {for all}} \quad 1 \le M \le N \end{aligned}$$

as a function of ${\boldsymbol{{\mathcal {I}}}}$ evaluated at ${\boldsymbol{{\mathcal {I}}}}^0$.

##### Remark 2.21

An explicit expression for $\det \langle H_M \rangle$ can be obtained by expanding it with respect to the last column and the $\iota _m$-th row:

2.30 $$\begin{aligned} \det \langle H_M \rangle = \sum _{m=1}^n \pm f_{\iota _m,\mathcal {I}_M} \det (H_{\iota _m}) \end{aligned}$$

Here $H_{\iota _m}$ is obtained from *H* by removing the last column and the $\iota _m$-th row. When there is a single input, i.e. $N=1$, the gradient $\nabla x_{o}$ reduces to ordinary derivative $x_o'$ and (2.28) gives the formula for $x_o'$ obtained in Madeira and Antoneli (2022). When there is a single input and a single input node, $N=n=1$, there is only one matrix $H_{\iota _m}=H$, called the *homeostasis matrix* and (2.28) gives the corresponding formula for $x_o'$ obtained in Wang et al. (2021). $\Diamond$

##### Definition 2.22

Let $\mathcal {G}$ be a multiple inputs network. The *core subnetwork* $\mathcal {G}_{c}$ of $\mathcal {G}$ is the subnetwork whose nodes are: (i) the input nodes $\iota _{1}, \ldots , \iota _{n}$, (ii) the regulatory nodes $\rho$ that are upstream from the output node and downstream of at least one input node, and (iii) the output node *o*. The arrows of $\mathcal {G}_{c}$ are the arrows of $\mathcal {G}$ connecting the nodes of $\mathcal {G}_{c}$. $\Diamond$

From this point we will depart from Madeira and Antoneli (2024) and incorporate the alternative approach from the previous section 2.2.1. Let $\mathcal {G}$ be a core multiple inputs network with inputs $\mathcal {I}_{1}, \ldots , \mathcal {I}_{N}$. The $\mathcal {I}_{M}$*-specialized network* $\mathcal {G}_{\mathcal {I}_{M}}$ is defined as the (single input prameter) input-output network consisting of the network $\mathcal {G}$ with the input nodes being exactly the nodes affected by the input parameter ${\mathcal {I}}_M$. In principle, the specialized network $\mathcal {G}_{\mathcal {I}_{M}}$ is a multiple input node network with single input parameter $\mathcal {I}_M$, as studied in Madeira and Antoneli (2022) and considered in the previous section 2.2.1. Now, when the specialized network $\mathcal {G}_{\mathcal {I}_{M}}$ has more than one input node we can apply the construction of Theorem 2.19 and replace $\mathcal {G}_{\mathcal {I}_{M}}$ by its augmented version $\mathcal {G}^\diamond _{\mathcal {I}_{M}}$. In this way all specialized networks are single-input single output networks.

However, it may happen that, although the multiple inputs network ${\mathcal {G}}$ is core, when one drops all the other input parameters, the network $\mathcal {G}_{\mathcal {I}_{M}}$ (or $\mathcal {G}^\diamond _{\mathcal {I}_{M}}$) become non-core. Therefore, one needs to consider the corresponding core networks $\mathcal {G}^{c}_{\mathcal {I}_{M}}$ (or $\mathcal {G}^{\diamond \, c}_{\mathcal {I}_{M}}$). Moreover, some of these single-input single-output networks might have the input the same as the output node. In any case, the algorithm can deal with all these networks.

Now, in order to put together the results of computation of the homeostasis subnetworks for each network $\mathcal {G}_{\mathcal {I}_{M}}$ (or $\mathcal {G}^\diamond _{\mathcal {I}_{M}}$) we need one more definition.

##### Definition 2.23

The *vector determinant* associated to an input-output network is the vector-valued function defined by

2.31 $$\begin{aligned} \widehat{h}= \big (\det \langle H_{\mathcal {I}_1} \rangle , \det \langle H_{\mathcal {I}_2} \rangle , \ldots , \det \langle H_{\mathcal {I}_N} \rangle \big ), \end{aligned}$$

where $\det \langle H_{\mathcal {I}_{M}} \rangle$, $M=1,\ldots ,N$, is the determinant of generalized homeostasis matrix of the network $\mathcal {G}_{\mathcal {I}_{M}}^c$ or its augmented version $\left( \mathcal {G}^\diamond _{\mathcal {I}_{M}}\right) ^c$, according with the situation. The vector-valued function $\widehat{h}$ can be considered as a (formal) polynomial mapping on the ‘variables’ $f_{j,x_i}$ and $f_{j,I_M}$. $\Diamond$

The following theorem ensures that $\widehat{h}$ contains all the information to classify the homeostasis subnetworks (see Madeira and Antoneli (2024)).

##### Theorem 2.24

The irreducible factors of $\widehat{h}$ are exactly the irreducible factors of $\nabla x_o$ that can induce homeostasis.

The König-Frobenius theorem (Schneider 1977; Brualdi and Cvetkoić 2009) (see also Wang et al. (2021); Madeira and Antoneli (2022)) imply that the components of the polynomial mapping $\widehat{h}$ can be factorized as the product of the determinants of the irreducible diagonal blocks of each $\langle H_{\mathcal {I}_M} \rangle$ (defined up to row and column permutations). An irreducible block *B* of some $\langle H_{\mathcal {I}_M} \rangle$ is called a *homeostasis block*. We can further collect the factors that are common irreducible diagonal blocks of all matrices $\langle H_{\mathcal {I}_M} \rangle$ and bring them to the front as scalar factors. Then we can write

2.32 $$\begin{aligned} \widehat{h} = \det (B_1) \cdots \det (B_k) \, \left( \prod _{j_1}\det (B_{\mathcal {I}_1}^{j_1}), \ldots , \prod _{j_N}\det (B_{\mathcal {I}_N}^{j_N}) \right) \end{aligned}$$

Therefore, we can split the problem of classifying homeostasis types supported by $\mathcal {G}$ into two cases according to whether the components of $\widehat{h}$ have a common scalar factor or not.

##### Definition 2.25

Let $\mathcal {G}$ be a core multiple inputs network and consider its vector determinant $\widehat{h}$ (2.32). A homeostasis block corresponding to scalar factor $\det (B_i)$ of $\widehat{h}$ is called a *pleiotropic homeostasis block*. The other homeostasis blocks are called *coincidental*. $\Diamond$

##### Remark 2.26

In genetics, pleiotropy refers to the phenomenon when a single locus affects multiple traits. Here, we employed the term *pleiotropic homeostasis* referring to the fact that the vanishing of one single homeostasis block leads to the vanishing of the whole homeostasis vector $\widehat{h}$. $\Diamond$

##### Definition 2.27

Let $\mathcal {G}$ be a core multiple inputs network.

- We say that *pleiotropic homeostasis* occurs when at least one pleiotropic block has vanishing determinant at some fixed input value. The pleiotropic blocks determine the *pleiotropic homeostasis types* of $\mathcal {G}$, which can be of appendage, structural or counterweight types.
- We say that *coincidental homeostasis* occurs when a *N*-tuple of coincidental blocks $\big (B_{\mathcal {I}_1}^{j_1},\ldots , B_{\mathcal {I}_N}^{j_N}\big )$ has simultaneously vanishing determinants at some fixed input value. The *N*-tuples of coincidental blocks determine the *coincidental homeostasis types* of $\mathcal {G}$. Coincidental blocks can be of appendage, structural or counterweight types. Hence, the coincidental homeostasis type associated to a *N*-tuple of coincidental blocks is a combination of single homeostasis types. $\Diamond$

### Reduction to the Single Input Node Case

As discussed above, although the algorithm for the classification of homeostasis subnetworks is formulated for single input node networks, the other cases can be reduced to this case, as far as the classification of homeostasis subnetworks is concerned. That is, in applying the algorithm, one does not need to specify the detailed dynamics (knowing at least one such dynamics exists is enough); only the network structure (i.e., the adjacency matrix or edge list) is needed for the algorithm to determine the classification.

Networks with multiple input nodes, ${\mathcal {G}}$, can be readily adapted to the single input node case by augmenting the original network to ${\mathcal {G}}^\diamond$ through the introduction of a new input node that connects to all original input nodes (Theorem 2.19). For multiple inputs networks, one can apply the algorithm repeatedly by considering one input parameter at a time, augmenting each specialized network with a new input node if necessary, and then combine the results together as explained above. The reduction of the specialized networks to their core network is done automatically by the algorithm (see Section 3). As shown before, this step is necessary to obtain only the subnetworks corresponding to the irreducible factors of the vector determinant.

## Algorithm Overview

We develop an automated procedure for identifying and classifying all homeostasis subnetworks in input-output networks, based on the theoretical framework introduced in Section 2. This automated classification algorithm operates on a single user-defined *input data file*, with a special structure, that encodes the network topology (represented as a directed graph) together with the designation of input and output nodes. To specify the network topology, users may provide either an admissible system associated with the network structure, the corresponding adjacency matrix, or an edge list specifying network connectivity.

### Remark 3.1

The algorithm is designed for single-input-single-output networks with single input node. For networks with multiple input nodes, users must first reformulate the network into an equivalent single-input-node representation, as described in Subsection 2.2.1 (Theorem 2.19). For networks with multiple inputs, users must provide separate input data files for each designated input parameter and run the algorithm independently for each case, then combine the resulting outputs, as described in Subsection 2.2.2. We illustrate this procedure using concrete examples in the following section. $\Diamond$

Given an input data file defining an input-output network, the algorithm identifies all homeostasis subnetworks ${\mathcal {K}}_\eta$, including appendage homeostasis subnetworks and structural homeostasis subnetworks, as outlined in algorithm 2.1.2. For each ${\mathcal {K}}_\eta$, the algorithm derives the corresponding homeostasis condition $\det (B_\eta )=0$, where $B_\eta$ is an irreducible diagonal block $B_\eta$ of the homeostasis matrix *H* for the full input-output network.

**Fig. 1:**
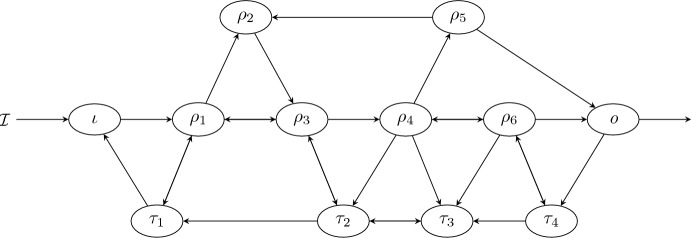
Network Diagram of the 12-node example, adapted from Wang et al. (2021)

Below, we illustrate the workflow of the algorithm and its typical outputs using a 12-node example network (Fig. 1) whose homeostatic structure has been fully analyzed in Wang et al. (2021). All tables and figures in this section, except Fig. 1, are generated directly from the algorithm.

### 12-node Network Example

In the 12-node network example (Fig. 1), input node $\iota$ receives a single input $\mathcal {I}$ and node *o* is the output node. For computational purposes, the algorithm represents network nodes with integer IDs. In this example, nodes are assigned IDs 0 through 11, as summarized in Table 1.

**Table 1:** Mapping between internal node IDs and original node labels for the 12-node example

Node ID	Node Label	Node ID	Node Label
0	$\iota$ (input node)	6	$\rho _6$
1	$\rho _1$	7	$\tau _1$
2	$\rho _2$	8	$\tau _2$
3	$\rho _3$	9	$\tau _3$
4	$\rho _4$	10	$\tau _4$
5	$\rho _5$	11	*o* (output node)

**Fig. 2:**
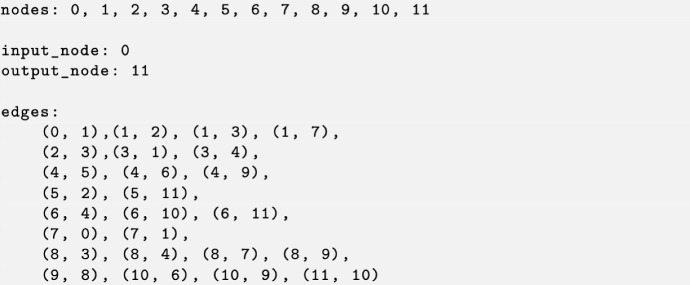
Input data file for the 12-node network example

An input data file for this network is shown in Fig. 2. The network structure is specified through an edge list representation, where a pair (*i*, *j*) represents an arrow from node *i* to *j*. The first line lists the node IDs used in the network, while the next two lines designate the input and output nodes.

**Fig. 3:**
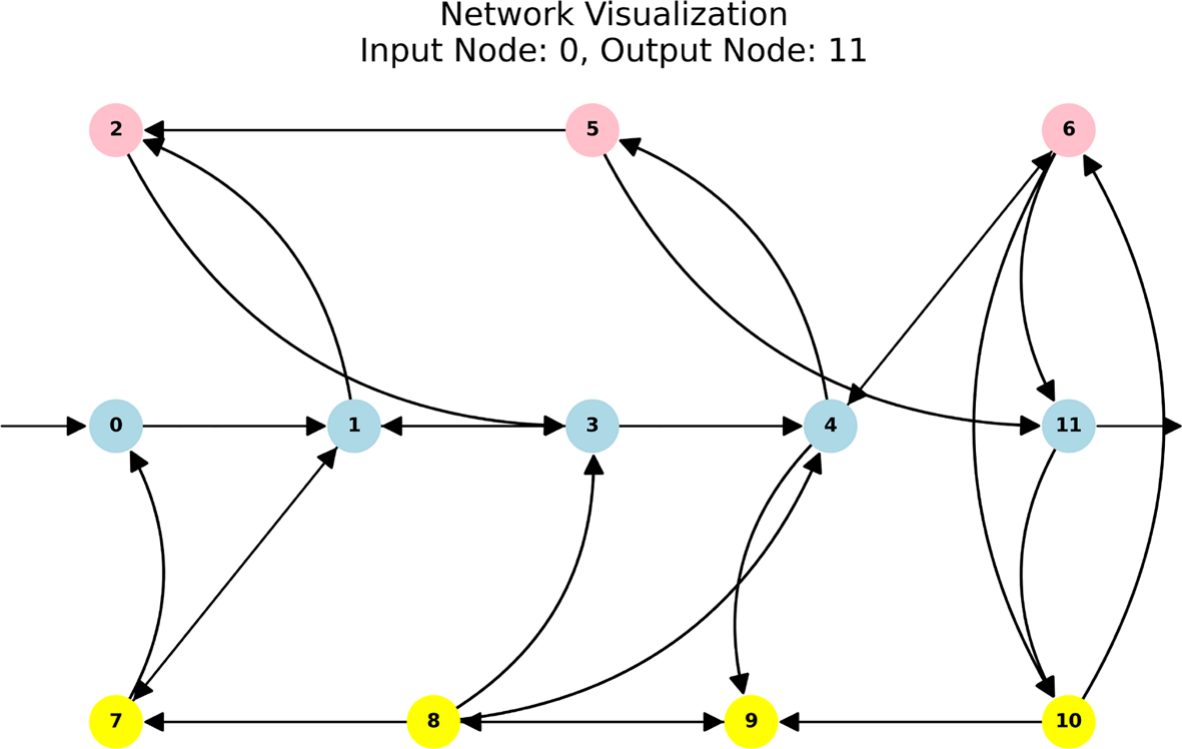
Algorithm-generated core network for the 12-node example from Fig. 1. Blue: super-simple nodes; pink: simple but non-super-simple nodes; yellow: appendage nodes (Color figure online)

Given this input file, the algorithm constructs the corresponding input-output network and extracts the *core network*, as shown in Fig. 3.

Users may optionally provide a label mapping in the input data file to associate each node ID with a user-defined node label (e.g., see Fig. 8 for an input data file of the Cholesterol example in Subsection 4.1). When such a mapping is supplied, all exported tables and network visualizations are generated using these labels (see Fig. 9).

### Simple paths and node categories

**Fig. 4:**
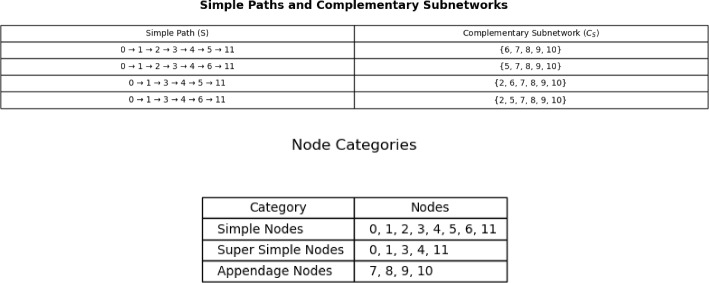
Algorithm-generated tables of simple paths, complementary subnetworks, and node categories for the 12-node example. In the node categories table, super-simple nodes are arranged in downstream order: $0>1>3>4>11$

Within the core input-output network (Fig. 3), the algorithm enumerates all $\iota o$*-simple paths* from the designated input node (node 0) to the designated output node (node 11), together with their associated *complementary subnetworks*. Based on these outputs, nodes are classified into *super-simple nodes*, *simple nodes*, and *appendage nodes*. The *appendage subnetwork* ${\mathcal {A}}_{\mathcal {G}}$ consisting of all appendage nodes and their induced edges is also constructed. These outputs are returned by the algorithm in tabular form; some are shown in Fig. 4.

#### Remark 3.2

In all algorithm-generated network visualizations (e.g., Fig. 3), blue nodes denote super-simple nodes, pink nodes denote simple but not super-simple nodes, and yellow nodes denote appendage node. $\Diamond$

In addition to network visualizations and tables, the algorithm writes its textual output to an automatically generated file, run_output.txt. In what follows, boxed blue text reproduces selected excerpts from the output file for the 12-node example.

### Appendage homeostasis subnetworks

As a first step in classifying homeostasis subnetworks, the algorithm determines the *appendage path components* from the appendage subnetwork ${\mathcal {A}}_{\mathcal {G}}$: 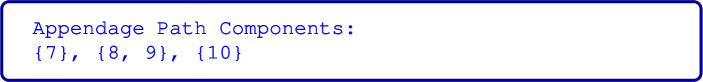


Among these components, those satisfying the no-cycle condition are classified as the appendage homeostasis subnetworks $\mathcal {A}_j$. 
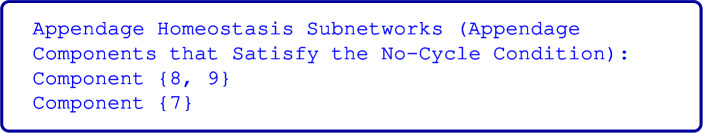


**Fig. 5:**
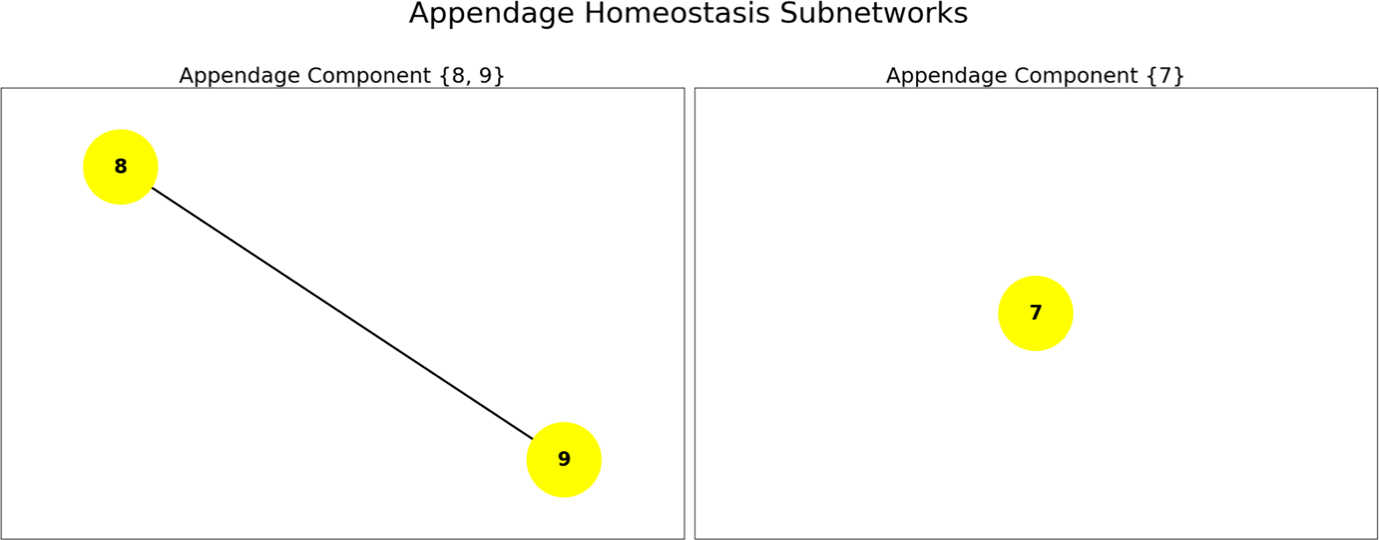
Algorithm-generated appendage homeostasis subnetworks for the 12-node example

These homeostasis subnetworks are also returned as network visualizations, shown in Fig. 5.

### Structural homeostasis subnetworks

The algorithm next determines the structural homeostasis subnetworks by enumerating all *super-simple structural subnetworks* constructed from adjacent super-simple nodes. Since there are five super-simple nodes (Fig. 4), this yields a total of four structural homeostasis subnetworks: 
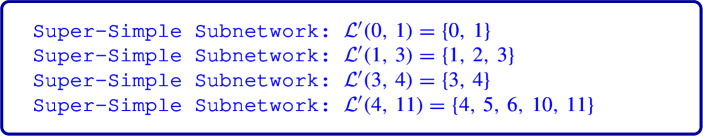

**Fig. 6:**
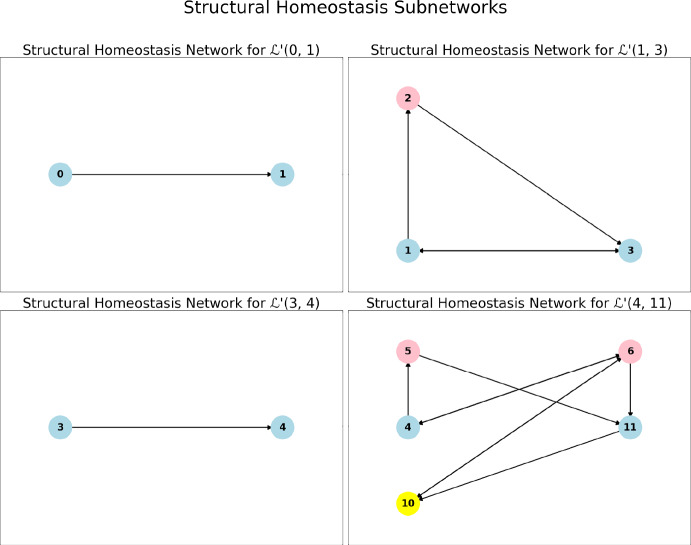
Algorithm-generated structural homeostasis subnetworks for the 12-node example

Visualizations of these structural homeostasis subnetworks are shown in Fig. 6.

**Fig. 7:**
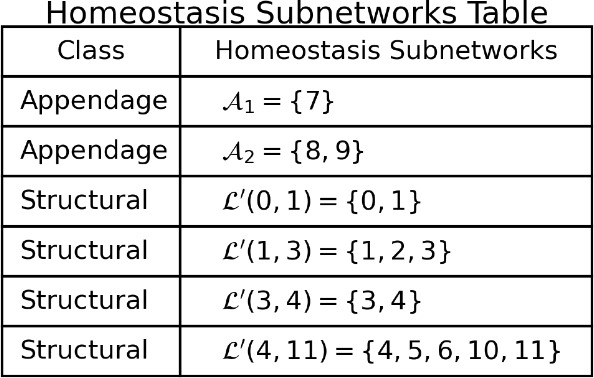
Algorithm-generated table of homeostasis subnetworks for the 12-node example

Finally, the algorithm returns a table summarizing all identified homeostasis subnetworks, shown in Fig. 7.

For each identified homeostasis subnetwork (${\mathcal {K}}_\eta$), the algorithm also outputs the associated irreducible homeostasis block ($B_\eta$) and saves them to run_output.txt, as listed below. The corresponding homeostasis condition is $\det (B_\eta )=0$ and $\det (B_\xi )\ne 0$ for all $\xi \ne \eta$. For example, to verify whether homeostasis in the 12-node example is induced by ${\mathcal {A}}_2$ (Fig. 7), one evaluates the corresponding matrix block at the equilibrium and checks whether its determinant $f_{8, x_8} f_{9, x_9} - f_{8, x_9} f_{9, x_8}$ vanishes at the homeostasis point. 
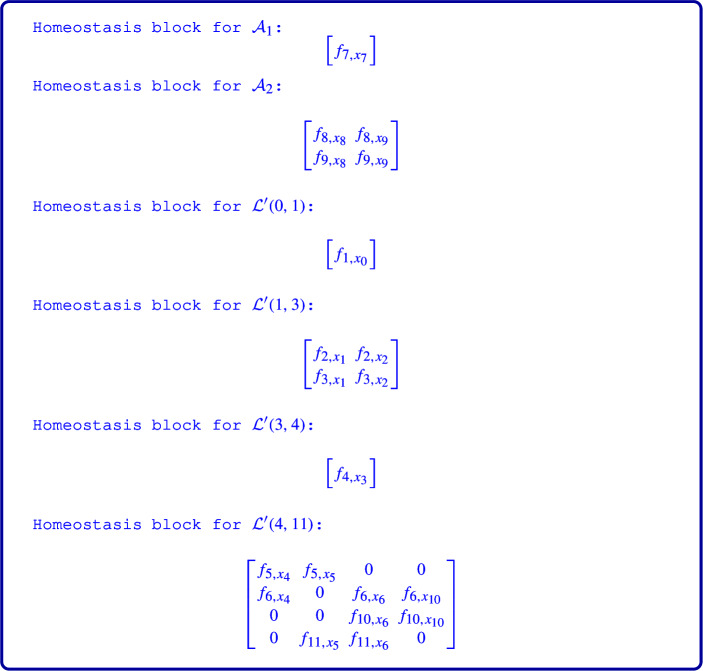


The results obtained by the algorithm for this example are fully consistent with the analytic classification reported in Wang et al. (2021), validating the accuracy of our algorithmic framework.

## Applications

In this section, we apply our algorithm to a series of biologically motivated network models, ranging from small to relatively large networks and covering both single-input and multiple-input network architectures. We consider both networks in which the input and output nodes are distinct and networks in which the input coincides the output. Our goal is to automatically identify all homeostasis motifs that would be difficult or impractical to detect manually, particularly in large networks. For networks of small to intermediate size, the algorithm results are validated against analytic classification results available from previous studies or carried out in the present work.

### Cholesterol network with single input node

**Fig. 8:**
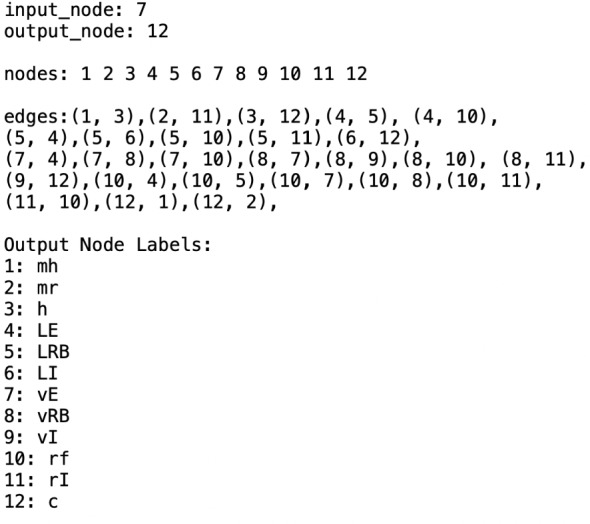
Input data file for the cholesterol network

Our first example is a model of intracellular cholesterol regulation proposed by Pool et al. (2018). This is a single-input-single-output network consisting of 12 nodes. In the corresponding *input data file* (Fig. 8), we assign IDs 1 through 12 to the network nodes (i.e., model variables), as summarized in Table 2.

**Table 2:** Node IDs, variable names and biological meanings for the cholesterol model. LDL: low density lipoprotein; VLDL: very low density lipoprotein

Nodes	Variables	Biological name	Nodes	Variables	Biological name
1	$m_h$	HMGCR mRNA	7	$v_E$ (input node)	free VLDL
2	$m_r$	LDLR mRNA	8	$v_{RB}$	receptor bound VLDL
3	*h*	HMGCR	9	$v_I$	internalised VLDL
4	$l_E$	free LDL	10	$r_f$	free unbound receptors
5	$l_{RB}$	receptor bound LDL	11	$r_I$	internalised receptors
6	$l_I$	internalised LDL	12	*c* (output node)	intracellular cholesterol

**Fig. 9:**
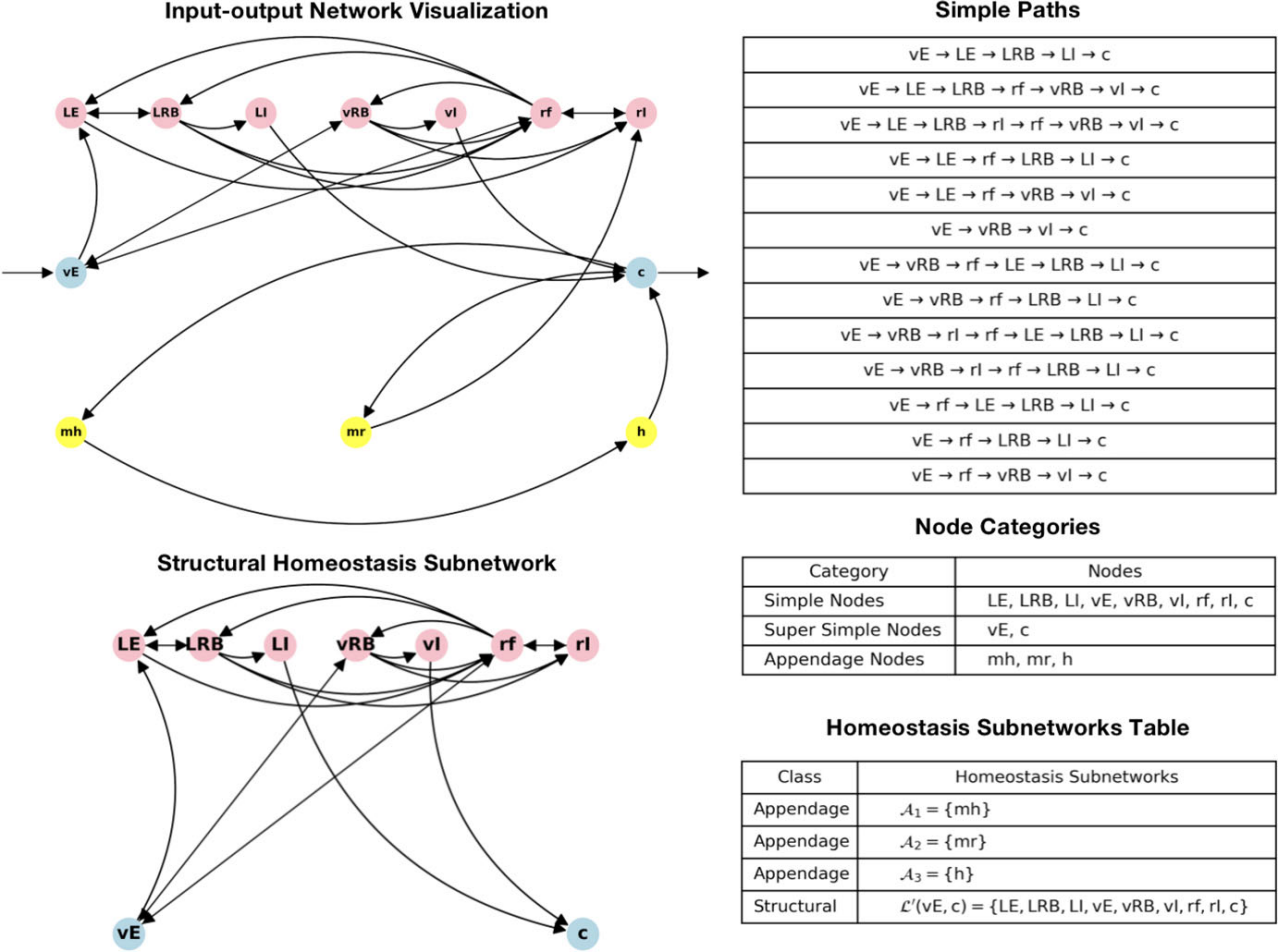
Algorithm-generated outputs for the cholesterol model. Blue: super-simple nodes; pink: simple but non-super-simple nodes; yellow: appendage nodes (Color figure online)

Based on the model formulation in Pool et al. (2018), we first write down the generic admissible system (see (A.1) in Appendix 1). The corresponding mathematical network structure can be encoded in the input data file using the admissible system, its adjacency matrix or edge list. Here we use an edge list representation (see Fig. 8). Below this list, a mapping from node IDs to their corresponding variable names is also included. The algorithm results are therefore reported using these labels rather than numeric node IDs (see Fig. 9).

Fig. 9 includes a visualization of the cholesterol network with node $v_E$ designated as the input node and node *c* as the output node, a table of $\iota o$-simple paths, a table of node categories, and the final classification table listing all identified homeostasis subnetworks. Consistent with the analytic classification results in Duncan et al. (2024), our algorithm identifies the same homeostasis subnetworks: one structural homeostasis motif ${\mathcal {L}}'\{v_E, c\}$ (see its network visualization in Fig. 9, lower left), and three appendage subnetworks, each consisting of a single appendage node.

To determine which of these mechanisms underlies homeostasis of the output node *c* in the full biochemical model, the corresponding homeostasis conditions ($\det (B_\eta )=0$) must be checked. The algorithm lists all the irreducible homeostasis blocks $B_\eta$ in the output file. We also provide them in Appendix A, Fig. 19.

#### Remark 4.1

For demonstration purposes, we consider admissible systems under the assumption that all variables in the original ODE system (in this example and all subsequent examples) are independent. Biochemical network models, however, frequently exhibit conservation laws or other algebraic constraints. When such constraints are present, the system should first be reduced to an equivalent ODE formulation involving only independent variables (see Oellerich et al. (2021); Jin and Rempala (2026)). For example, if a conservation relation of the form $x_1+x_2=C$ holds, where *C* is a constant, one eliminates a dependent variable (e.g., by substituting $x_1=C-x_2$) and writes the admissible system in terms of the remaining independent variables. The algorithm then applies to the reduced system without modification. $\Diamond$

### Chemotaxis network with multiple input nodes

The mathematical modeling of chemotaxis can be roughly divided into two types: single cell models and bacterial population models (Tindall et al. 2008b, a). Single cell models consider the activation of the flagellar motor by detection of attractants and repellents in the extracellular medium. The flagellar motor activity of bacteria is regulated by a signal transduction pathway, which integrates changes of environmental chemical concentrations into a behavioral response. Assuming mass-action kinetics, the reactions in the signal transduction pathway can be modeled mathematically by ODEs. The population models describe evolution of bacterial density by parabolic PDEs allowing movement up-the-gradient, the most prominent feature of chemotaxis.

Understanding the response of bacteria such as *E. coli* to external attractants has been the subject of experimental work and mathematical models for nearly 40 years. Many models of the chemotaxis have been formulated and developed to provide a comprehensive description of the cellular processes and include details of receptor methylation, ligand-receptor binding and its subsequent effect on the biochemical signaling cascade, along with a description of motor driving CheY/CheY-P levels, the main output of the chemotaxis system (see Tindall et al. (2008b) for a survey).

Here, we consider a minimal model for the *E. coli* response proposed by Clausznitzer et al. (2010); Edgington and Tindall (2015, 2018), which nevertheless is in good agreement with experimental findings. It has four variables for the concentrations of CheA/CheA-P ($a_p$), CheY/CheY-P ($y_p$), CheB/CheB-P ($b_p$) and the receptor methylation (*m*) and is given by the following system of ODEs (in non-dimensional form):

4.1 $$\begin{aligned} \begin{aligned}&\frac{d m}{d t} = \gamma _{R}\, (1 - \phi (m,L)) - \gamma _{B} \, \phi (m,L) \, b_{p}^{2} \\&\frac{d a_{p}}{d t} = \phi (m,L) \, k_{1} \, (1 - a_{p}) - k_{2} \, (1 - y_{p})\, a_{p} - k_{3} \, (1 - b_{p}) \, a_{p} \\&\frac{d y_{p}}{d t} = \alpha _{1} \, k_{2} \, (1 - y_{p})a_{p} - k_{4} \, y_{p} \\&\frac{d b_{p}}{d t} = \alpha _{2} \, k_{3} \, (1 - b_{p}) \, a_{p} - k_{5} \, b_{p} \end{aligned} \end{aligned}$$

where $\gamma _B$, $\gamma _R$, $k_1,\ldots ,k_5$ are non-dimensional parameters, the extracellular ligand concentration *L* is the *external parameter* and the function $\phi$ is determined by a Monod–Wyman–Changeux (MWC) description of receptor clustering

4.2 $$\begin{aligned} \phi (m,L) = \frac{1}{1 + e^{F(m,L)}} \qquad {\text {with}}\qquad F(m,L) = N\left[ 1 - \frac{m}{2} + \log \left( \frac{1 + \frac{L}{K_{a}^{\text {off}}}}{1 + \frac{L}{K_{a}^{\text {on}}}}\right) \right] \end{aligned}$$

The key observation of Edgington and Tindall (2018) is that system (4.1) has a unique asymptotically stable equilibrium $X^*=(m^*,a_p^*,y_p^*,b_p^*)$, with $a_p^*$, $y_p^*$ and $b_p^*$ positive and $m^{*}$ is a real number, for the non-dimensional parameters obtained from the parameter values originally used in Clausznitzer et al. (2010). Furthermore, Edgington and Tindall (2018) were able to show that some pairs of parameters might yield oscillatory behavior, but in regions of parameter space that are outside the experimentally relevant.

**Fig. 10:**
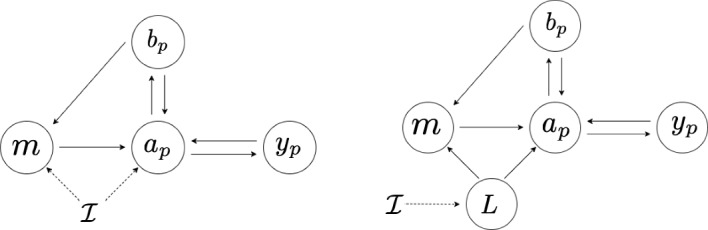
(Left ) Network ${\mathcal {G}}$ corresponding to the model equations (4.1) for *E. coli* chemotaxis. (Right) Augmented network ${\mathcal {G}}^\diamond$ obtained from ${\mathcal {G}}$

The network corresponding to the model equations (4.1) has two input nodes *m* and $a_p$ (Fig. 10, left). In Table 3 we list the variables of model (4.1) and the corresponding nodes in the network. As described in Remark 3.1, in order to apply the algorithm, one must first convert this multiple-input-node network into a single-input-node representation. To do so, we introduce a new input node *L* and connect it to both *m* and $a_p$. This converts the original multiple-input-node network *G* to the augmented single-input-node network $G^\diamond$ (Fig. 10, right). By Theorem 2.19, this reformulation preserves both the homeostasis matrix and the homeostasis subnetworks of the original network system.

**Table 3:** Node IDs, variable names and biological meanings for the chemotaxis model

Nodes	Variables	Biological name
1	*L* (input node)	Extracellular ligand
2	*m*	Receptor methylation
3	$a_p$	CheA/CheA-P
4	$b_p$	CheB/CheB-P
5	$y_p$ (output node)	CheY/CheY-P

**Fig. 11:**
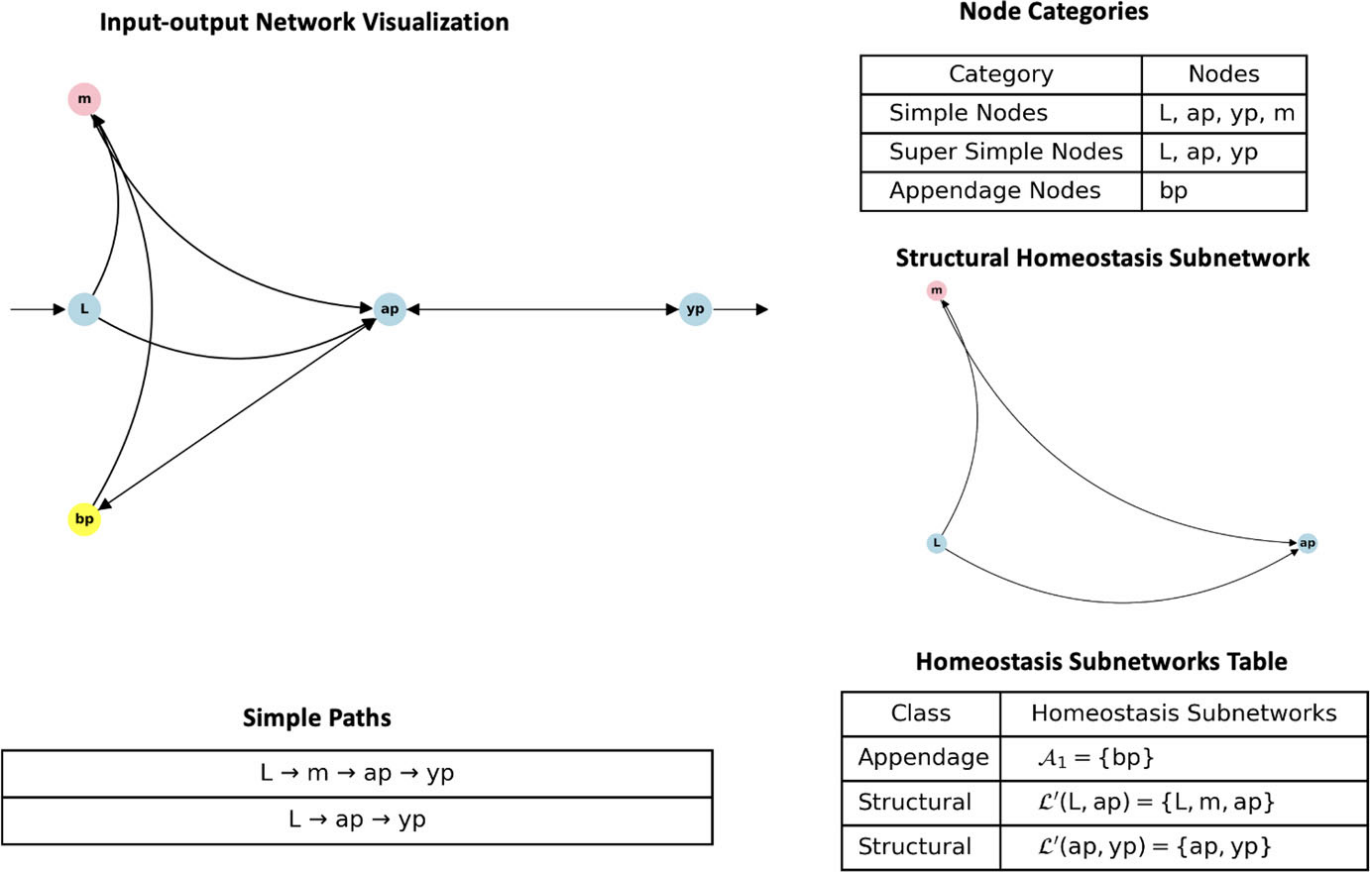
Algorithm-generated outputs for the chemotaxis model. Blue: super-simple nodes; pink: simple but non-super-simple nodes; yellow: appendage nodes (Color figure online)

Representative outputs obtained by the classification algorithm applied to the augmented network $G^\diamond$ are shown in Fig. 11, with a layout similar to that in Fig. 9. The algorithm identifies three homeostasis mechanisms, summarized in the lower-right table. Among them is a structural homeostasis motif, ${\mathcal {L}}'\{L, a_p\}=\{L,m,a_p\}$, arising from the balance between the two paths $L\rightarrow m\rightarrow a_p$ and $L\rightarrow a_p$. A visualization of this motif is shown on the right. The other two mechanisms correspond to substrate inhibition along the edge $a_p\rightarrow y_p$ and null-degradation on the appendage node $b_p$. Going back to the model equations (4.1) and using the homeostasis condition returned by the algorithm (not shown), it can be checked that the structural homeostasis motif, ${\mathcal {L}}'\{L, a_p\}$, is the one responsible for the homeostasis observed in this model.

In the original approach of Madeira and Antoneli (2022) the homeostasis subnetworks are computed by hand, using the method described in the paper. The result of the calculation is that there are three types of homeostasis: (1) appendage (null-degradation) homeostasis associated with $b_p$, (2) structural (Haldane) homeostasis associated with $a_p\rightarrow y_p$ and (3) input counterweight homeostasis associated with $m \rightarrow a_p$. This is consistent with the result obtained here. The input counterweight homeostasis subnetwork $m \rightarrow a_p$ corresponds to the structural block ${\mathcal {L}}'\{L, a_p\}=\{L,m,a_p\}$, when the node *L* is removed from the network.

### Dopamine network with multiple input nodes

Best et al. (2009); Nijhout et al. (2014) proposed a mathematical model to study homeostasis of extracellular dopamine (eDA) in response to variations in the activities of the enzyme tyrosine hydroxylase (TH) and the dopamine transporters (DATs). This model is given by a system of differential equations describing the underlying biochemical network (see, Best et al. (2009), Figure 1). Their modeling and simulation results show that eDA remains approximately constant over a range of TH and DAT activities, indicating the capability of the network to maintain eDA homeostasis.

**Table 4:** Mapping of node IDs to variable names and biological meanings for the dopamine model

Nodes	Variables	Biological name	Nodes	Variables	Biological name
1	TH (input node)	Tyrosine hydroxylase	6	cDa	Cytosolic dopamine
2	bh2	Dihydrobiopterin	7	vDa	Vesicular dopamine
3	bh4	Tetrahydrobiopterin	8	DAT	Dopamine transporter
4	tyr	Tyrosine	9	tyrpool	The tyrosine pool
5	L-dopa	3,4-dihyroxyphenylalanine	10	eDa (output node)	Extracellular dopamine

**Fig. 12:**
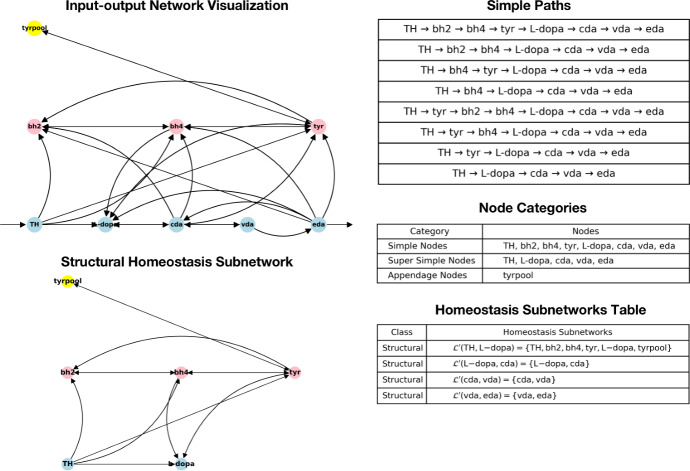
Algorithm-generated outputs for the dopamine model. Blue: super-simple nodes; pink: simple but non-super-simple nodes; yellow: appendage nodes (Color figure online)

Here, we focus on the case in which TH activity is treated as the input parameter and apply our algorithm to identify homeostatic mechanisms embedded in the full biological network structure. Since variations in TH activity influence four variables, bh2, bh4, tyr and L-dopa, the corresponding network is a single-input-single-output network *G* with multiple input nodes. As discussed in Subsection 4.2, applying the algorithm requires converting *G* into an augmented network $G^\diamond$ by introducing a new input node TH, and connecting it to nodes bh2, bh4, tyr and L-dopa. The core network of $G^\diamond$ is shown in Fig. 12 (upper left). Note that the node DAT is excluded from the core network. Table 4 lists the model variables, their biological names, and the corresponding nodes in $G^\diamond$.

Representative outputs obtained from applying the algorithm to $G^\diamond$ are shown in Fig. 12, with a similar layout as in Fig. 9. For a general admissible system respecting the structure of the dopamine biological network, infinitesimal homeostasis of eDA can arise through four distinct mechanisms, summarized in the lower-right table. Three of these correspond to substrate inhibition along edge L-dopa $\rightarrow$ cDa, cDa $\rightarrow$ vDa, or vDA $\rightarrow$ eDa. The fourth homeostasis mechanism arises through a larger structural subnetwork $\mathcal {L}'\{\text {TH},\text {L-dopa}\}$ consisting of nodes TH, bh2, bh4, tyr, L-dopa and tyrpool. A visualization of this subnetwork is displayed in the lower-left panel of Fig. 12. The homeostasis blocks corresponding to these four mechanisms are provided in Appendix B.1, Fig. 20.

To validate the outputs of our algorithm, we also compute the Jacobian matrix and the homeostasis matrix *H* of the dopamine admissible system (B.1), and directly determine the irreducible factors of $\det (H)$ whose vanishing corresponds to homeostasis mechanisms (see Appendix B.2). These results are consistent with the algorithm outputs.

### Hepatic one-carbon metabolism coupled with methionine, choline and betaine synthesis: multiple inputs and multiple choices of output nodes

In this subsection, we apply the algorithm to a 17-node input-output biochemical network underlying the model of hepatic one-carbon metabolism proposed in Sadre-Marandi et al. (2018). We examine homeostasis mechanisms in choline (cho) and homocysteine (hcy) under variations in input parameters methionine and methylenetetrahydrofolate reductase (MTHFR) activity. This network contains two distinct input parameters and two possible output nodes. The remaining state variable (network node) names and their biological meanings are listed in Table 5.

**Table 5:** Mapping of node IDs to variable names and biological meanings for the hepatic one-carbon metabolism model

Nodes	Variables	Biological name	Nodes	Variables	Biological name
1	met	Methionine	10	mthf	5-methyltetrahydrofolate
2	sam	S-adenosylmethionine	11	gnmt	Glycine N-methyltransferase
3	sah	S-adenosylhomocysteine	12	gnmtf	GNMT-5mTHF
4	hcy	Homocysteine	13	fgnmtf	5mTHF-GNMT-5mTHF
5	dhf	Dihydrofolate	14	bet	Betaine
6	thf	Tetrahydrofolate	15	bet-ald	Betaine aldehyde
7	fthf	10-formyltetrahydrofolate	16	cho	Choline
8	ch	5,10-methenyltetrahydrofolate	17	pc	PtCho
9	ch2	5,10-methylenetrahydrofolate			

**Fig. 13:**
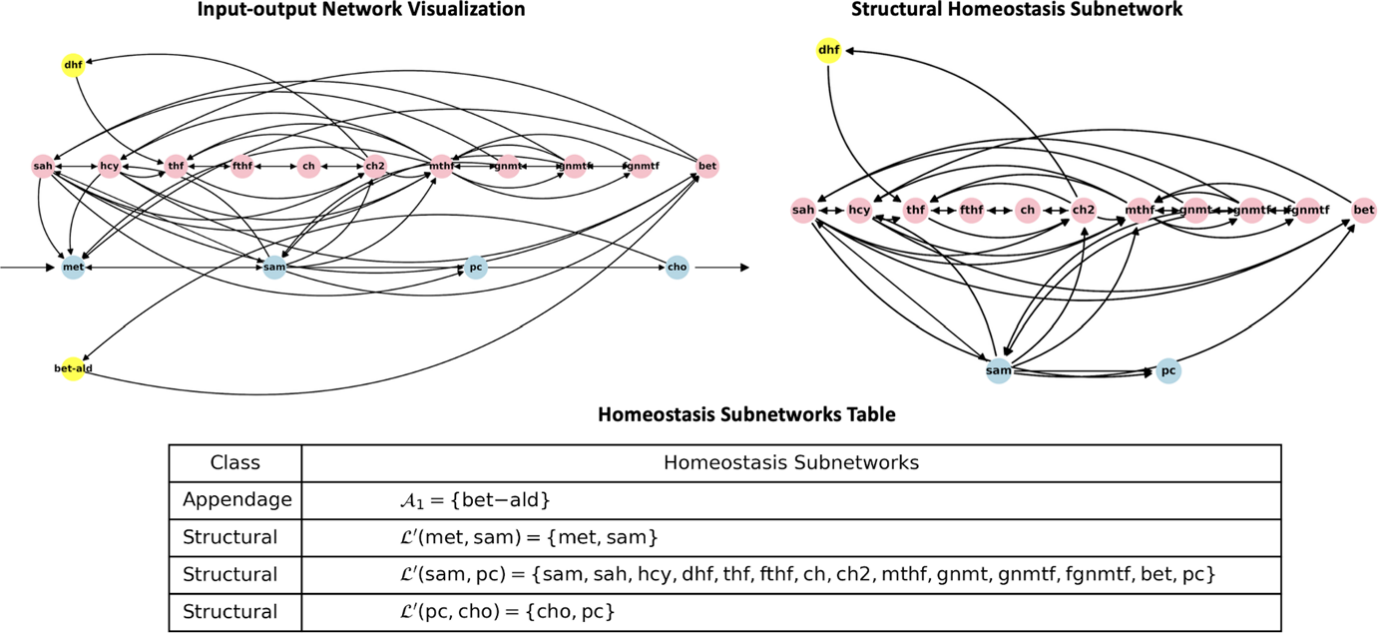
Algorithm-generated outputs for the one-carbon metabolism model with input node met and output node cho. Blue: super-simple nodes; pink: simple but non-super-simple nodes; yellow: appendage nodes. There are 54 $\iota o$-simple paths in total

**Fig. 14:**
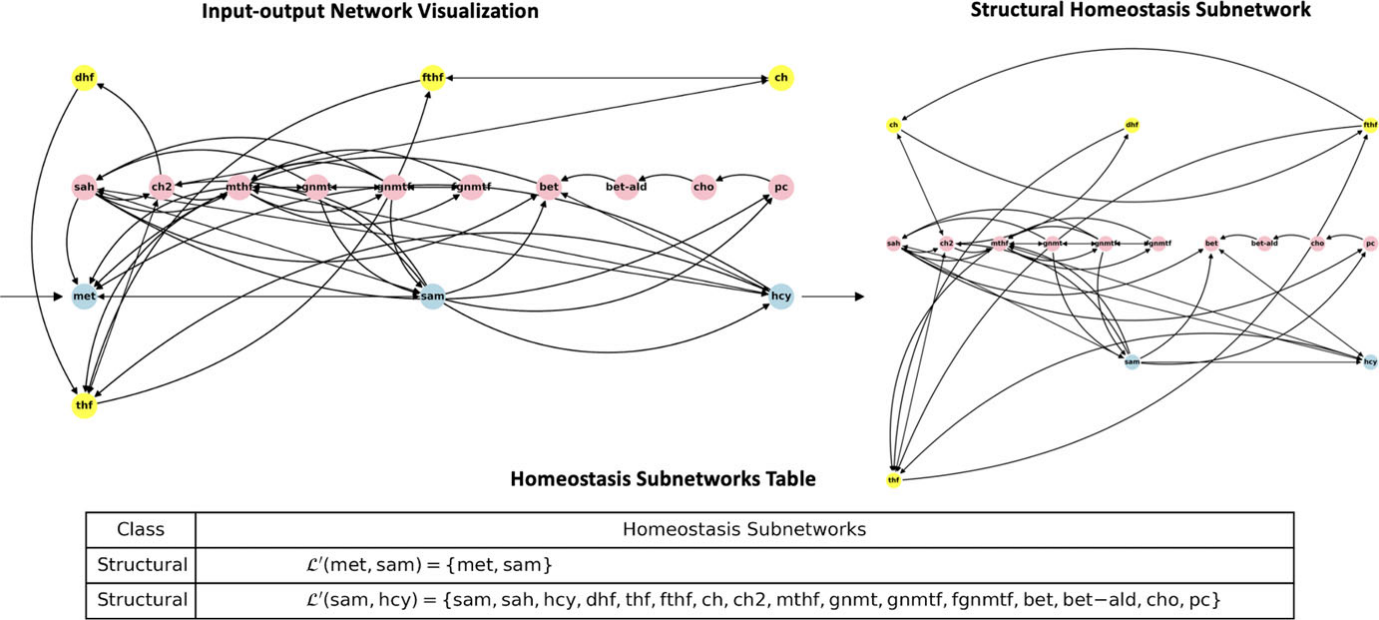
Algorithm-generated outputs for the one-carbon metabolism model with input node met and output node hcy, with a total of 45 $\iota o$-simple paths. Blue: super-simple nodes; pink: simple but non-super-simple nodes; yellow: appendage nodes (Color figure online)

**Fig. 15:**
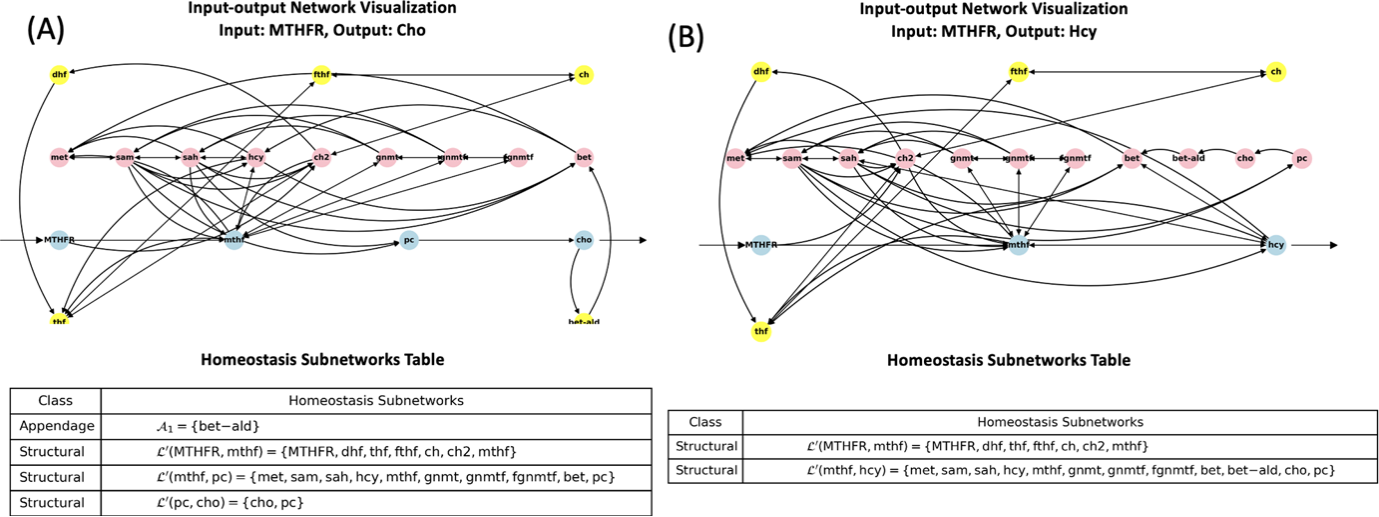
Algorithm-generated outputs for the one-carbon metabolism model with the input node MTHFR and output node (A) cho and (B) hcy. Blue: super-simple nodes; pink: simple but non-super-simple nodes; yellow: appendage nodes (Color figure online)

Variations in the first input methionine only affects a single input node met. We can therefore directly apply our algorithm and classify all homeostasis mechanisms with either cho or hcy as the output node. Representative outputs for these cases are shown in Figs. 13 and 14. In contrast, the other input parameter, MTHFR activity, influences two state variables ch2 and mthf. This leads to a multiple-input node network *G* and, as discussed previously, can be handled by introducing a new input node denoted as MTHFR and adding edges from this node to ch2 and mthf (see the augmented input-output networks $G^\diamond$ in Fig. 15).

When met is the input node and cho is the output (Fig. 13), there are 54 $\iota o$-simple paths. The algorithm identifies four homeostasis mechanisms: one appendage subnetwork consisting of a single homeostasis node bet-ald, and three structural homeostasis subnetworks. Two of the structural mechanisms arise from substrate inhibition along either the edge met$\rightarrow$sam or the edge pc$\rightarrow$cho. The third is a 14-node structural homeostasis subnetwork $\mathcal {L}'\{\texttt {sam},\texttt {pc}\}$, whose structure is displayed in Fig. 13 (upper right).

When met is the input node and hcy the output (Fig. 14), there are 45 $\iota o$-simple paths. In this case, the algorithm detects two structural homeostasis mechanisms. Infinitesimal homeostasis in hcy can arise either via substrate inhibition along met$\rightarrow$sam, or through a 16-node structural subnetwork $\mathcal {L}'\{\texttt {sam},\texttt {hcy}\}$, whose network structure is displayed in Fig. 14 (upper right).

When the input node is MTHFR and output node is cho, the algorithm detects four homeostasis mechanisms (see Fig. 15A): one appendage and three structural. In particular, two blocks (${\mathcal {A}}_1=\{\texttt {bet-ald}\}$ and ${\mathcal {L}}'\{\texttt {pc, cho}\}$) are identical to those identified when met is the input node. These correspond to pleiotropic homeostasis types, in which the vanishing of either block leads to homeostasis in cho with respect to both methionine input and MTHFR activity (see Definition 2.25). By contrast, when MTHFR is the input node and hcy is the output node, the algorithm identifies two structural homeostasis mechanisms (see Fig. 15B). Neither coincides with those obtained when met is the input node. Therefore, in this case there is only coincidental homeostasis.

### Hepatic one carbon metabolism coupled with glutathione synthesis pathway: moderate size network with multiple input node choices

In this subsection, we apply the algorithm to a 34-node input-output biochemical network underlying the model of hepatic one-carbon metabolism coupled with the glutathione synthesis pathway proposed in Reed et al. (2008). This is an extension of the model for hepatic mitochondrial folate metabolism from Nijhout et al. (2006), designed to include cysteine and glutathione metabolism. This mathematical model is quite complicated, since it includes all of one carbon metabolism and not just the transsulfuration pathway in order to couple with the glutathione synthesis pathway.

**Fig. 16:**
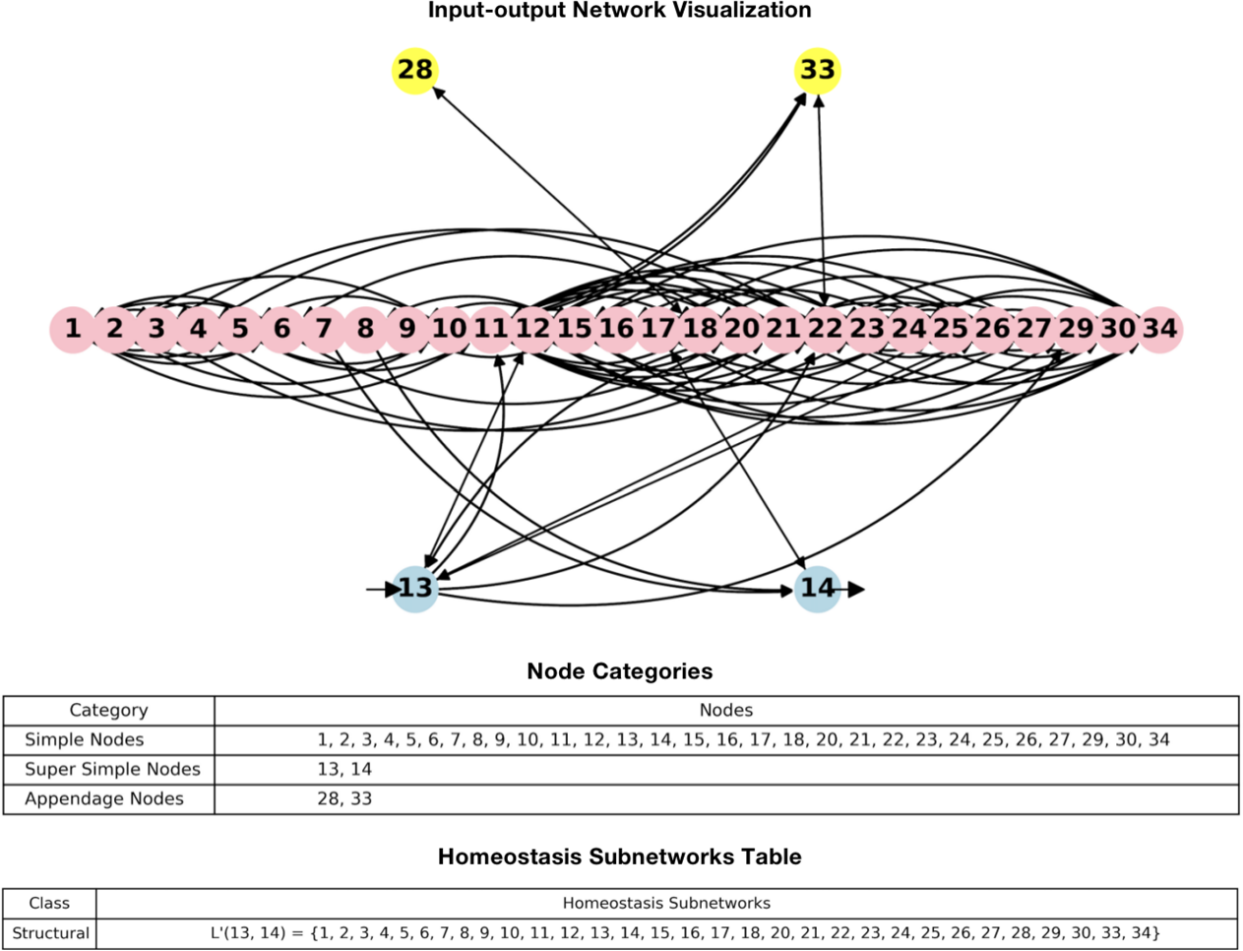
Algorithm-generated outputs for the glutathione model with Input Node 13. Blue: super-simple nodes; pink: simple but non-super-simple nodes; yellow: appendage nodes (Color figure online)

**Fig. 17:**
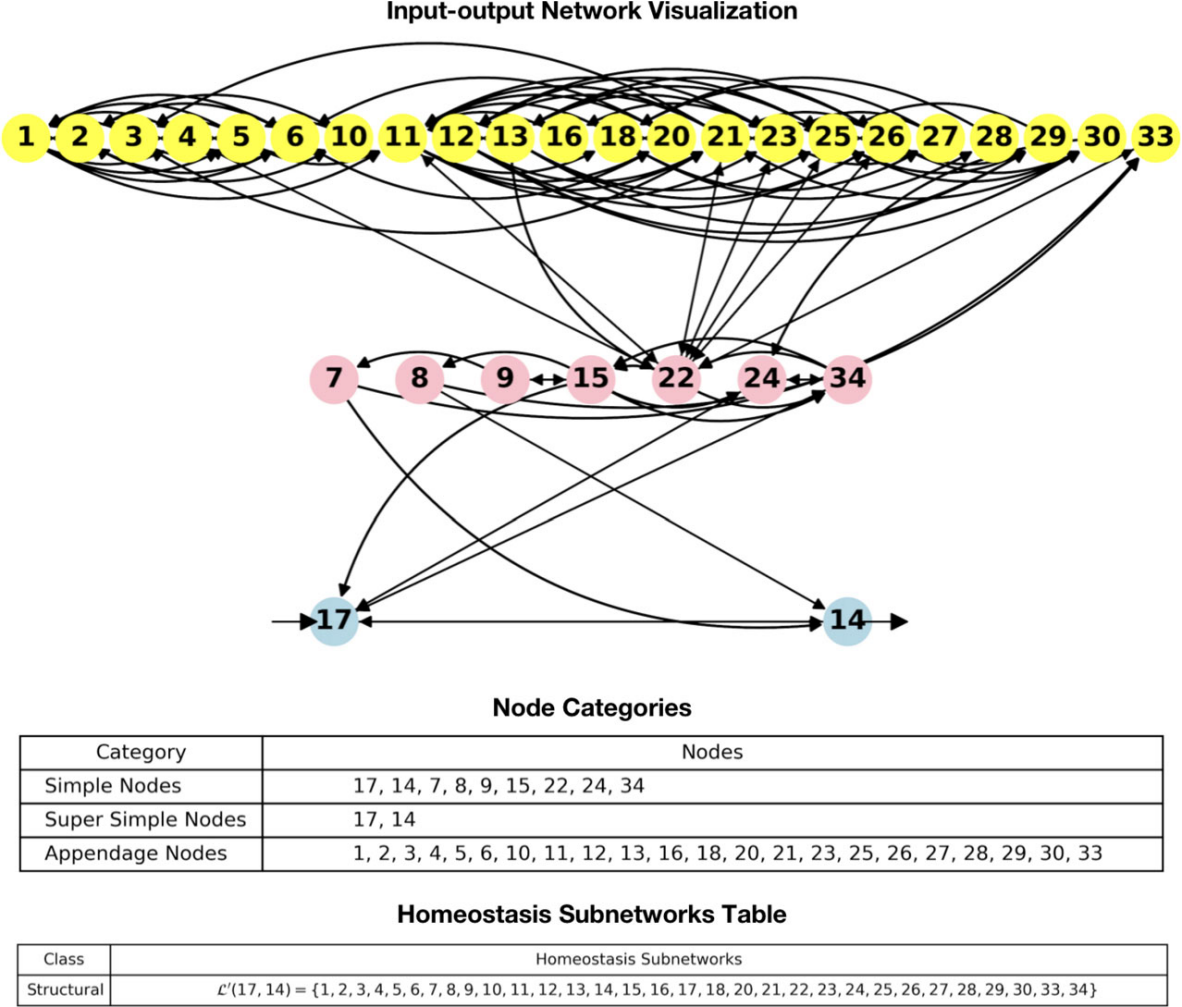
Algorithm-generated outputs for the glutathione model with Input Node 17. Blue: super-simple nodes; pink: simple but non-super-simple nodes; yellow: appendage nodes (Color figure online)

The 34 variables (nodes) are listed in Table 6. The output node is node 14 (bglu). We consider two choices of input node and input parameter:

- Node 13 (5mf). Here the input parameter ${\mathcal {I}}$ is total concentration of cellular folate (one carbon metabolism), see Figure 16 for the corresponding algorithm outputs.
- Node 17 (cglu). Here, the input parameter ${\mathcal {I}}$ is the total concentration of cellular glutamate (glutamate synthesis pathway), see Figure 17 for the corresponding altorighm outputs.

In both cases, there is only one single homeostatic mechanism, given by a large structural block that involves all nodes in the core network.

#### Remark 4.2

In Figs. 16 and 17, due to the large size and density of the network, many arrows overlap and are therefore not clearly visible. In both cases, node 19 (bcys), node 31 (src), and node 32 (dmg) are excluded from the core network. $\Diamond$

**Table 6:** Mapping of node IDs to variable names and biological meanings for the glutathione model

Nodes	Variables	Biological name	Nodes	Variables	Biological name
1	mthf	tetrahydrofolate	18	c10f	10-formyltetrahydrofolate
2	m10f	10-formyltetrahydrofolate	19	bcys	blood cysteine
3	mser	mitochondrial serine	20	cCOO	cytosolic formate
4	mgly	mitochondrial glycine	21	cser	cytosolic serine
5	m2cf	5-10-methylenetetrahydrofolate	22	cgly	cytosolic glycine
6	mCOO	mitochondrial formate	23	c2cf	5-10-methylenetetrahydrofolate
7	bgsg	blood glutathione disulfide	24	ccys	cytosolic cysteine
8	bgsh	blood glutathione	25	sam	S-adenosylmethionine
9	cgsg	cytosolic glutathione disulfide	26	sah	S-adenosylhomocysteine
10	m1cf	5-10-methenyltetrahydrofolate	27	c1cf	5-10-methenyltetrahydrofolate
11	cthf	tetrahydrofolate	28	aic	P-ribosyl-5-amino-4-imidazole carboxamide
12	hcy	homocysteine	29	met	methionine
13	5mf (input node 1)	5-methyltetrahydrofolate	30	cyt	cystathionine
14	bglu (output node)	blood glutamate	31	src	sarcosine
15	cgsh	cytosolic glutathione	32	dmg	dimethylglycine
16	dhf	dihydrofolate	33	bgly	blood glycine
17	cglu (input node 2)	cytosolic glutamate	34	glc	glutamyl-cysteine

### Zinc homeostasis: network with input=output

Zinc is an essential micro-nutrient for plants, because it plays an important role in many enzymes catalyzing vital cellular reactions. In higher doses, however, zinc is toxic. Therefore, plants have to strictly control and adjust the uptake of zinc through their roots depending on its concentration in the surrounding soil. This is achieved by a complicated control system consisting of sensors, transmitters and zinc transporter proteins.

In Claus et al. (2015) the authors propose a mathematical model for regulation of zinc uptake in roots of *Arabidopsis thaliana* based on the uptake of zinc, expression of a transporter protein and the interaction between an activator and inhibitor. The equations in Claus et al. (2015) are obtained by an *ad hoc* dimensional reduction of a model proposed in Claus and Chavarría-Krauser (2012).

The model of Claus et al. (2015) is given by a system of four nonlinear ordinary differential equations. For convenience we represent the concentration of each component by: $x_1=[\mathrm {Zn^{2+}}]$, $x_2=[\textrm{mRNA}]$, $x_3=[\textrm{ZIP}]$ and $x_4=[\textrm{Dimer}]$. That is, $x_1,\ldots ,x_4$ are state variables and the equations are

4.3 $$\begin{aligned} \begin{aligned} \dot{x}_1&= \mathcal {I}x_4 - c_1 x_1 \\ \dot{x}_2&= 1 - a_2 x_2 x_3 - c_2 x_2\\ \dot{x}_3&= a_3 x_1 (v_1 - x_3) - a_5 x_2 x_3 - c_3 x_3 \\ \dot{x}_4&= a_4 x_2^3 (v_2 - x_4) - c_4 x_4 \end{aligned} \end{aligned}$$

Here, $a_i$, $c_j$ and $v_l$ are positive parameters. The quantity of interest to be controlled is the *concentration of intracellular zinc* ($x_1=[\textrm{Zn}^{2+}]$). There is one external control parameter, $\mathcal {I}$, representing the *(normalized) concentration of extracelullar zinc*.

**Fig. 18:**
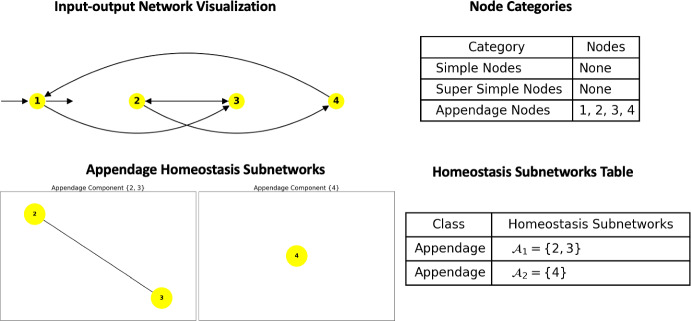
Algorithm-generated outputs for the zinc model. This input-output network contains only appendage nodes (yellow nodes) (Color figure online)

**Fig. 19:**
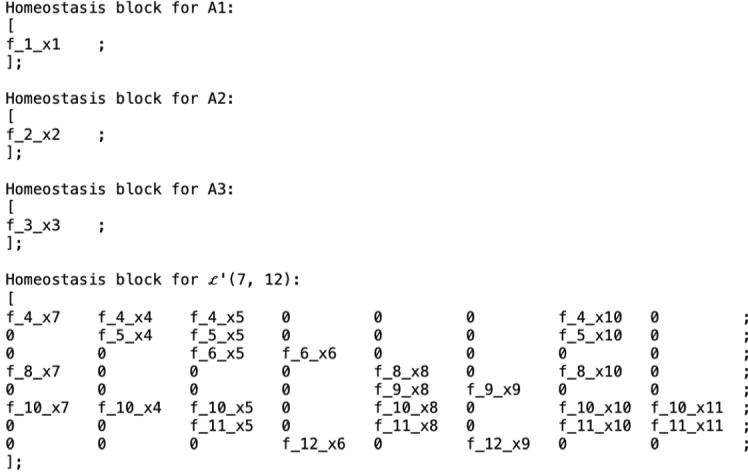
Algorithm output showing homeostasis blocks for the cholesterol example (Fig. 9), with the node ID-variable name mapping provided in Table 2

**Fig. 20:**
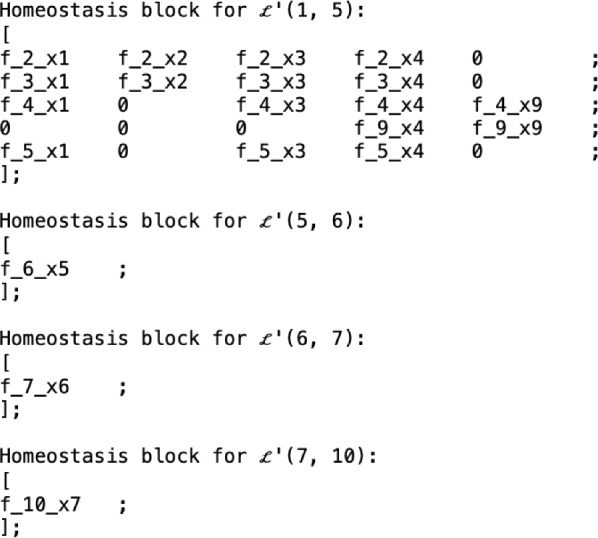
Algorithm output showing homeostasis blocks for the dopamine example in Fig. 12, with the node ID-variable name mapping provided in Table 4

Thus, this network is an example of input=output (Fig. 18, upper left). Consistent with the theory in Subsection 2.1.1, the algorithm detects no simple paths, simple nodes or super-simple nodes; all nodes are appendage nodes. The algorithm identifies two appendage homeostasis mechanisms, summarized in the lower-right table in Fig. 18: the 2-node appendage subnetwork  (${\mathcal {A}}_1$) and null-degradation on node 4 (${\mathcal {A}}_2$). These results agree with the analytic classification in Antoneli et al. (2025).

## Conclusion and Outlook

Despite intensive studies on the structural properties of networks that achieve homeostasis or perfect adaptation (Ma et al. 2009; Ang and McMillen 2013; Tang and McMillen 2016; Ferrell 2016; Qian and Vecchio 2018; Araujo and Liota 2018; Vecchio et al. 2018; Aoki et al. 2019; Hong et al. 2025; Khammash 2021; Yi et al. 2000; Xiao and Doyle 2018; Cappelletti et al. 2020; Briat et al. 2016; Araujo and Liotta 2023; Qiao et al. 2019; Golubitsky and Wang 2020; Golubitsky et al. 2020; Wang et al. 2021), the mathematical complexity of these results can make them difficult to apply in practice, thereby limiting their broader accessibility and usability. In this paper we present a Python-based algorithm that automates the identification of homeostasis subnetworks of an input-output network. More specifically, given an input–output network specified solely by its connectivity structure (e.g., an edge list, adjacency matrix, or admissible ODE system) and the designation of input and output nodes, the algorithm automatically computes the relevant graph-theoretical structures and enumerates all homeostasis subnetworks. The algorithm is able to handle several classes of input-output networks, including those with a single input parameter (with single or multiple input nodes), multiple input parameters, and cases when the input and output nodes coincide. For systems subject to conservation laws or other algebraic constraints (Jin and Rempala 2026; Oellerich et al. 2021), the algorithm remains applicable after reformulating the admissible system in terms of independent variables when such constraints can be identified.

We illustrate the algorithm by applying it to several biological examples, covering all the cases mentioned above. We also included some examples of moderately large networks to showcase the algorithm’s capability to handle network sizes that are beyond what can be done by hand.

The wide applicability of the algorithm, is due to a new method, developed in this paper, to reduce the analysis of networks with multiple input nodes to the case of a single input node, by introducing an augmented single-input representation (see Theorem 2.19). This new result, together with the observation from Madeira and Antoneli (2024) that the analysis of a multiple inputs network amounts to deal with one parameter at a time, allowed us to extend the scope of an algorithm designed to treat the single input node/parameter case to other classes of networks.

The new theoretical approach introduced in this paper may be helpful in the development of another algorithm to enumerate the homeostasis patterns of an input-output network. A *homeostasis pattern* is defined as a set of nodes, in addition to the output node, that are simultaneously infinitesimally homeostatic. It has been shown in Duncan et al. (2024); Antoneli et al. (2026b) that to each homeostasis subnetwork there corresponds a unique (generic) homeostasis pattern. Moreover, it is shown that the classification of homeostasis patterns is reduced to a combinatorial problem. Hence, it is expected that there is a corresponding algorithm for the computation of homeostasis patterns.

So far, the theoretical framework developed for infinitesimal homeostasis (Golubitsky and Wang 2020; Golubitsky et al. 2020; Wang et al. 2021; Madeira and Antoneli 2022, 2024; Antoneli et al. 2025) has focused on deterministic network systems. Jin and Rempala (2026) extended the notion of infinitesimal homeostasis to mass-action systems with a specialized graph structure. In this context, they consider deterministic systems and their associated stochastic version, where the input parameter is a conservation-law constant. In the stochastic case, the output is the expectation of the stationary distribution of an output random variable. They show that the condition for infinitesimal homeostasis in terms of the homeostasis determinant, that is $\det (H)=0$, is the same in both cases. This implies, in particular, that the algorithm described here gives a meaningful answer when applied to the deterministic cases, as well as, the stochastic. However, a general classification of homeostasis types and a topology-based characterization of stochastic homeostasis mechanisms remain undeveloped. Extending our graph-theoretic framework to stochastic settings, especially when the input itself is modeled as noise, would be an interesting direction for future work. The relevance of our algorithm to any possible stochastic extension of the deterministic theory is linked to the following question: does this kind of stochasticity preserve the condition for infinitesimal homeostasis in terms of the homeostasis determinant?

## Data Availability

The implementation of the algorithm, together with input data files, algorithm outputs for all network examples used in this work, and documentation, is publicly available at GitHub: https://github.com/Homeostasis-Classification/Homeostasis-Classification-Algorithm.

## Appendices

In all examples presented in Section 4, we reported the homeostasis subnetworks detected by the algorithm, denoted as ${\mathcal {K}}_\eta$, but did not list the associated homeostasis blocks $B_\eta$. In the appendix sections below, we include the homeostasis blocks, as recorded in the output file run_output.txt, for the cholesterol network in Appendix A and for the dopamine network in Appendix B.

In addition, we perform in Appendix B an analytic classification of the homeostasis mechanisms for the dopamine network. This is obtained by computing the Jacobian matrix *J*, the homeostasis matrix *H*, and factorizing $\det (H)$ to identify all irreducible homeostasis blocks, thereby verifying the accuracy of the algorithm outputs.

For the remaining examples, we omit listing the homeostasis blocks here; they can be found in the corresponding run_output.txt files provided in the GitHub repository (see Code Availability).

## Appendix A Cholesterol network

The admissible system for the Cholesterol network equations proposed in Pool et al. (2018) is given by the following equations:

A.1 $$\begin{aligned} \frac{dx_1}{dt}&= f_1\!\left( x_{1}, x_{12}\right) \nonumber \\ \frac{dx_2}{dt}&= f_2\!\left( x_{2}, x_{12}\right) \nonumber \\ \frac{dx_3}{dt}&= f_3\!\left( x_{1}, x_{3}\right) \nonumber \\ \frac{dx_4}{dt}&= f_4\!\left( x_{4}, x_{5}, x_{7}, x_{10}\right) \nonumber \\ \frac{dx_5}{dt}&= f_5\!\left( x_{4}, x_{5}, x_{10}\right) \nonumber \\ \frac{dx_6}{dt}&= f_6\!\left( x_{5}, x_{6}\right) \nonumber \\ \frac{dx_7}{dt}&= f_7\!\left( x_{7}, x_{8}, x_{10}\right) \nonumber \\ \frac{dx_8}{dt}&= f_8\!\left( x_{7}, x_{8}, x_{10}\right) \nonumber \\ \frac{dx_9}{dt}&= f_9\!\left( x_{8}, x_{9}\right) \nonumber \\ \frac{dx_{10}}{dt}&= f_{10}\!\left( x_{4}, x_{5}, x_{7}, x_{8}, x_{10}, x_{11}\right) \nonumber \\ \frac{dx_{11}}{dt}&= f_{11}\!\left( x_{2}, x_{5}, x_{8}, x_{10}, x_{11}\right) \nonumber \\ \frac{dx_{12}}{dt}&= f_{12}\!\left( x_{3}, x_{6}, x_{9}, x_{12}\right) \end{aligned}$$

where $x_i$ denotes the state variable associated with nodes *i*, with the mapping between nodes, variables, and their biological names given in Table 2.

As discussed in Subsection 4.1, the algorithm detects three appendage and one structural homeostasis subnetworks (Fig. 9, lower-right table). Their corresponding homeostasis blocks, which are irreducible components of the homeostasis matrix *H*, are listed in Fig. 19.

## Appendix B Dopamine network

In this appendix section, we provide additional details for the dopamine network example discussed in Subsection 4.3.

### Appendix B.1 Algorithm-detected homeostasis blocks

We consider a model of dopamine synthesis and release proposed by Best et al. (2009), where the activity of the enzyme tyrosine hydroxylase (TH) is the *external input parameter*. Table 4 lists the model variables and the corresponding nodes in the network. Variation of TH activity affects variables bh2, bh4, tyr and L-dopa, making this an input-output network with multiple input nodes. In order to apply the algorithm, one must first convert this network *G* into a augmented single-input-node network $G^\diamond$ by introducing a new input node TH and connecting it to bh2, bh4, tyr and L-dopa (Remark 3.1 and Theorem 2.19).

As discussed in Subsection 4.3, the algorithm detects four structural homeostasis subnetworks (Fig. 12, lower-right table). Their corresponding homeostasis blocks are listed in Fig. 20.

### Appendix B.2 Verification of algorithm outputs via direct computation

Below, we compute the Jacobian and homeostasis matrices for the admissible system of the augmented network $G^\diamond$, determine the irreducible factors of the homeostasis matrix *H*, and compare them with the blocks detected by the algorithm.

The admissible system of $G^\diamond$ is given by:

B.1 $$\begin{aligned} \begin{aligned} \frac{dx_1}{dt}&= f_1(x_1, {\mathcal {I}})\\ \frac{dx_2}{dt}&= f_2(x_1, x_2, x_3, x_4, x_6, x_{10})\\ \frac{dx_3}{dt}&= f_3(x_1, x_2, x_3, x_4, x_6, x_{10})\\ \frac{dx_4}{dt}&= f_4(x_1, x_3, x_4, x_6, x_9, x_{10})\\ \frac{dx_5}{dt}&= f_5(x_1, x_3, x_4, x_5, x_6, x_{10})\\ \frac{dx_6}{dt}&= f_6(x_5, x_6, x_7, x_8, x_{10})\\ \frac{dx_7}{dt}&= f_7(x_6, x_7)\\ \frac{dx_8}{dt}&= f_8(x_8)\\ \frac{dx_9}{dt}&= f_9(x_4, x_9)\\ \frac{dx_{10}}{dt}&= f_{10}(x_7, x_8, x_{10})\\ \end{aligned} \end{aligned}$$

where $x_i$ denotes the state variable associated with node *i* (see Table 4 for the corresponding model variables). In particular, $x_1$ denotes TH and represents the newly introduced input node influencing $x_2,\cdots , x_5$ (i.e., bh2, bh4, tyr, and L-dopa). Since node 8 is not downstream of the input node 1 and hence not part of the core network, it can be excluded when computing the homeostasis blocks.

The Jacobian matrix of (B.1), excluding $x_8$, is given by

$$\begin{aligned} J=\left[ \begin{array}{ccccccccc} f_{1,x_1} & 0 & 0 & 0 & 0 & 0 & 0 & 0 & 0\\ f_{2,x_1} & f_{2,x_2} & f_{2,x_3} & f_{2,x_4} & 0 & f_{2,x_6} & 0 & 0 & f_{2,x_{10}}\\ f_{3,x_1} & f_{3,x_2} & f_{3,x_3} & f_{3,x_4} & 0 & f_{3,x_6} & 0 & 0 & f_{3,x_{10}}\\ f_{4,x_1} & 0 & f_{4,x_3} & f_{4,x_4} & 0 & f_{4,x_6} & 0 & f_{4,x_9} & f_{4,x_{10}}\\ f_{5,x_1} & 0 & f_{5,x_3} & f_{5,x_4} & f_{5,x_5} & f_{5,x_6} & 0 & 0 & f_{5,x_{10}}\\ 0 & 0 & 0 & 0 & f_{6,x_5} & f_{6,x_6} & f_{6,x_7} & 0 & f_{6,x_{10}}\\ 0 & 0 & 0 & 0 & 0 & f_{7,x_6} & f_{7,x_7} & 0 & 0\\ 0 & 0 & 0 & f_{9,x_4} & 0 & 0 & 0 & f_{9,x_9} & 0\\ 0 & 0 & 0 & 0 & 0 & 0 & f_{10,x_7} & 0 & f_{10,x_{10}} \end{array} \right] \end{aligned}$$

The homeostasis matrix is obtained by removing the first row (corresponding to the input node 1) and the last column (corresponding to the output node 10):

$$ H=\begin{aligned} \left[ \begin{array}{cccccccc} f_{2,x_1} & f_{2,x_2} & f_{2,x_3} & f_{2,x_4} & 0 & f_{2,x_6} & 0 & 0\\ f_{3,x_1} & f_{3,x_2} & f_{3,x_3} & f_{3,x_4} & 0 & f_{3,x_6} & 0 & 0\\ f_{4,x_1} & 0 & f_{4,x_3} & f_{4,x_4} & 0 & f_{4,x_6} & 0 & f_{4,x_9}\\ f_{5,x_1} & 0 & f_{5,x_3} & f_{5,x_4} & f_{5,x_5} & f_{5,x_6} & 0 & 0\\ 0 & 0 & 0 & 0 & f_{6,x_5} & f_{6,x_6} & f_{6,x_7} & 0\\ 0 & 0 & 0 & 0 & 0 & f_{7,x_6} & f_{7,x_7} & 0\\ 0 & 0 & 0 & f_{9,x_4} & 0 & 0 & 0 & f_{9,x_9}\\ 0 & 0 & 0 & 0 & 0 & 0 & f_{10,x_7} & 0 \end{array} \right] \end{aligned} $$

By cofactor expansion, we obtain

B.2 $$\begin{aligned} \begin{aligned} \det (H)&= \pm f_{6,x_5} f_{7,x_6} f_{10,x_7} \det (B_1) \end{aligned} \end{aligned}$$

where

$$ B_1= \left[ \begin{array}{ccccc} f_{2, x_{1}} & f_{2, x_{2}} & f_{2, x_{3}} & f_{2, x_{4}} & 0\\ f_{3, x_{1}} & f_{3, x_{2}} & f_{3, x_{3}} & f_{3, x_{4}} & 0\\ f_{4, x_{1}} & 0 & f_{4, x_{3}} & f_{4, x_{4}} & f_{4, x_{9}}\\ 0 & 0 & 0 & f_{9, x_{4}} & f_{9, x_{9}}\\ f_{5, x_{1}} & 0 & f_{5, x_{3}} & f_{5, x_{4}} & 0 \end{array}\right] . $$

The four irreducible factors obtained above ($f_{6,x_5},\,f_{7,x_6},\,f_{10,x_7},\, B_1$) are the same as those produced by the algorithm (Fig. 20). They give rise to the four structural homeostasis subnetworks identified by the algorithm.

## References

[CR1] Andrade PPAC, Madeira JLO, Antoneli F (2022) Homeostatic mechanisms in biological systems. 1–31, arXiv:2202.11218. 10.48550/arXiv.2202.11218

[CR2] Ang J, McMillen D (2013) Physical constraints on biological integral control design for homeostasis and sensory adaptation. Biophys J 104(2):505–515 23442873 10.1016/j.bpj.2012.12.015 PMC3552268

[CR3] Antoneli F, Golubitsky M, Stewart I (2018) Homeostasis in a feed forward loop gene regulatory network motif. J Theor Biol 445:103–109 29477558 10.1016/j.jtbi.2018.02.026

[CR4] Antoneli F, Golubitsky M, Jin J et al (2025) Homeostasis in input-output networks: Structure, classification and applications. Math Biosci 384:109435 40222590 10.1016/j.mbs.2025.109435

[CR5] Antoneli F, Best J, Golubitsky M et al (2026a) Homeostasis in networks with same input and output nodes and metal ion regulation. In preparation

[CR6] Antoneli F, Golubitsky M, Jin J et al (2026) Homeostasis in gene regulatory networks. Int J Biomath 19:2550070. 10.1142/S1793524525500706

[CR7] Aoki S, Lillacci G, Gupta A et al (2019) A universal biomolecular integral feedback controller for robust perfect adaptation. Nature 570:533–537 31217585 10.1038/s41586-019-1321-1

[CR8] Araujo R, Liota L (2018) The topological requirements for robust perfect adaptation in networks of any size. Nature Comm 9:1757 10.1038/s41467-018-04151-6 PMC5931626 29717141

[CR9] Araujo RP, Liotta LA (2023) Universal structures for adaptation in biochemical reaction networks. Nat Commun 14(1):2251 37081018 10.1038/s41467-023-38011-9 PMC10119132

[CR10] Best J, Nijhout HF, Reed MC (2009) Homeostatic mechanisms in dopamine synthesis and release: a mathematical model. Theor Biol Med Model 6(1):1–20 19740446 10.1186/1742-4682-6-21 PMC2755466

[CR11] Briat C, Gupta A, Khammash M (2016) Antithetic integral feedback ensures robust perfect adaptation in noisy biomolecular networks. Cell Syst 2(1):15–26 27136686 10.1016/j.cels.2016.01.004

[CR12] Brualdi R, Cvetkoić D (2009) A Combinatorial approach to matrix theory and its applications. Chapman & Hall/CRC Press

[CR13] Brualdi R, Ryser H (1991) Combinatorial matrix theory. Cambridge University Press, UK

[CR14] Cappelletti D, Gupta A, Khammash M (2020) A hidden integral structure endows absolute concentration robust systems with resilience to dynamical concentration disturbances. J R Soc Interface 17:171 10.1098/rsif.2020.0437 PMC7653391 33109021

[CR15] Claus J, Chavarría-Krauser A (2012) Modeling regulation of zinc uptake via ZIP transporters in yeast and plant roots. PLoS ONE 7(6):e37193. 10.1371/journal.pone.0037193 22715365 10.1371/journal.pone.0037193 PMC3371047

[CR16] Claus J, Ptashnyk M, Bohmann A et al (2015) Global Hopf bifurcation in the ZIP regulatory system. J Math Biol 71(4):795–816. 10.1007/s00285-014-0836-1 25312412 10.1007/s00285-014-0836-1

[CR17] Clausznitzer D, Oleksiuk O, Løvdok L et al (2010) Chemotactic response and adaptation dynamics in Escherichia coli. PLoS Comput Biol 6(5):1–11. 10.1371/journal.pcbi.1000784 10.1371/journal.pcbi.1000784 PMC2873904 20502674

[CR18] Drengstig T, Ni X, Thorsen K et al (2012) Robust adaptation and homeostasis by autocatalysis. J Phys Chem B 116(18):5355–5363 22506960 10.1021/jp3004568

[CR19] Duncan W, Antoneli F, Best J et al (2024) Homeostasis patterns. SIAM J Appl Dyn Syst 23(3):2262–2292

[CR20] Edgington MP, Tindall MJ (2015) Understanding the link between single cell and population scale responses of Escherichia coli in differing ligand gradients. Comp Struct Biotech J 13:528–538 10.1016/j.csbj.2015.09.003 PMC4660157 26693274

[CR21] Edgington MP, Tindall MJ (2018) Mathematical analysis of the Escherichia coli chemotaxis signalling pathway. Bull Math Biol 80(4):758–787 29404879 10.1007/s11538-018-0400-z PMC5862969

[CR22] Ferrell J (2016) Perfect and near perfect adaptation in cell signaling. Cell Syst 2:62–67 27135159 10.1016/j.cels.2016.02.006

[CR23] Golubitsky M, Stewart I (2017) Homeostasis, singularities and networks. J Math Biol 74:387–407 27255135 10.1007/s00285-016-1024-2

[CR24] Golubitsky M, Stewart I (2023) Dynamics and Bifurcation in Networks - Theory and Applications of Coupled Differential Equations. SIAM Philadelphia PA doi 10(1137/1):9781611977332

[CR25] Golubitsky M, Wang Y (2020) Infinitesimal homeostasis in three-node input-output networks. J Math Biol 80:1–23. 10.1007/s00285-019-01457-x 31919651 10.1007/s00285-019-01457-x

[CR26] Golubitsky M, Stewart I, Antoneli F, et al (2020) Input-output networks, singularity theory, and homeostasis. In: Advances in Dynamics, Optimization and Computation: A volume dedicated to Michael Dellnitz on the occasion of his 60th birthday. Springer, p 31–65

[CR27] Hong H, Moon S, Hirono Y et al (2025) Topological criterion for robust perfect adaptation of reaction fluxes in biological networks. Iscience 28(6):112394. 10.1016/j.isci.2025.112394 40530424 10.1016/j.isci.2025.112394 PMC12172989

[CR28] Jin J, Rempala GA (2026) Infinitesimal homeostasis in mass-action systems. J Math Biol 92(3):35. 10.1007/s00285-026-02352-y 41701291 10.1007/s00285-026-02352-y PMC12913314

[CR29] Khammash MH (2021) Perfect adaptation in biology Cell Syst 12(6):509–521 34139163 10.1016/j.cels.2021.05.020

[CR30] Lloyd A (2013) The regulation of cell size. Cell 154:1194 24034244 10.1016/j.cell.2013.08.053

[CR31] Ma W, Trusina A, El-Samad H et al (2009) Defining network topologies that can achieve biochemical adaptation. Cell 138:760–773 19703401 10.1016/j.cell.2009.06.013 PMC3068210

[CR32] Madeira JLO, Antoneli F (2022) Homeostasis in networks with multiple input nodes and robustness in bacterial chemotaxis. J Nonlinear Sci 32(3):37. 10.1007/s00332-022-09793-x

[CR33] Madeira JLO, Antoneli F (2024) Homeostasis in networks with multiple inputs. J Math Biol 89:17. 10.1007/s00285-024-02117-5 38902549 10.1007/s00285-024-02117-5 PMC11190020

[CR34] Nijhout H, Reed M (2014) Homeostasis and dynamic stability of the phenotype link robustness and plasticity. Integr Comp Biol 54(2):264–75 24729131 10.1093/icb/icu010

[CR35] Nijhout H, Reed M, Budu P et al (2004) A mathematical model of the folate cycle: new insights into folate homeostasis. J Biol Chem 279:55008–55016 15496403 10.1074/jbc.M410818200

[CR36] Nijhout H, Best J, Reed M (2014) Escape from homeostasis. Math Biosci 257:104–110 25242608 10.1016/j.mbs.2014.08.015

[CR37] Nijhout H, Best J, Reed M (2015) Using mathematical models to understand metabolism, genes and disease. BMC Biol 13:79 26400419 10.1186/s12915-015-0189-2 PMC4580265

[CR38] Nijhout H, Best J, Reed M (2018) Systems biology of robustness and homeostatic mechanisms. WIREs Syst Biol Med 11(3):e1440 10.1002/wsbm.1440 30371009

[CR39] Nijhout HF, Reed MC, Lam SL et al (2006) In silico experimentation with a model of hepatic mitochondrial folate metabolism. Theor Biol Med Model 3(1):40 17150100 10.1186/1742-4682-3-40 PMC1713227

[CR40] Nijhout HF, Sadre-Marandi F, Best J et al (2017) Systems biology of phenotypic robustness and plasticity. Integr Comp Biol 57(2):171–184 28859407 10.1093/icb/icx076

[CR41] Oellerich T, Emelianenko M, Liotta L et al (2021) Biological networks with singular jacobians: their origins and adaptation criteria. bioRxiv 2021.03.01.433197. 10.1101/2021.03.01.433197

[CR42] Pool F, Sweby PK, Tindall MJ (2018) An integrated mathematical model of cellular cholesterol biosynthesis and lipoprotein metabolism. Processes 6(8):134

[CR43] Qian Y, Vecchio DD (2018) Realizing ‘integral control’ in living cells: how to overcome leaky integration due to dilution? J R Soc Interface 15(139):20170902 29436515 10.1098/rsif.2017.0902 PMC5832733

[CR44] Qiao L, Zhao W, Tang C et al (2019) Network topologies that can achieve dual function of adaptation and noise attenuation. Cell Syst 9(3):271–285 31542414 10.1016/j.cels.2019.08.006 PMC6768439

[CR45] Reed M, Lieb A, Nijhout H (2010) The biological significance of substrate inhibition: a mechanism with diverse functions. BioEssays 32(5):422–429 20414900 10.1002/bies.200900167

[CR46] Reed M, Best J, Golubitsky M et al (2017) Analysis of homeostatic mechanisms in biochemical networks. Bull Math Biol 79(9):1–24 28884446 10.1007/s11538-017-0340-z PMC5842936

[CR47] Reed MC, Thomas RL, Pavisic J et al (2008) A mathematical model of glutathione metabolism. Theor Biol Med Model 5(1):8 18442411 10.1186/1742-4682-5-8 PMC2391141

[CR48] Sadre-Marandi F, Dahdoul T, Reed MC et al (2018) Sex differences in hepatic one-carbon metabolism. BMC Sys Biol 12(1):89 10.1186/s12918-018-0621-7 PMC6201565 30355281

[CR49] Schneider H (1977) The concepts of irreducibility and full indecomposability of a matrix in the works of frobenius, könig and markov. Lin Alg Appl 18:139–162

[CR50] Tang ZF, McMillen DR (2016) Design principles for the analysis and construction of robustly homeostatic biological networks. J Theor Biol 408:274–289 27378006 10.1016/j.jtbi.2016.06.036

[CR51] Thom R (1969) Topological models in biology. Topology 8(3):313–335. 10.1016/0040-9383(69)90018-4

[CR52] Thom R (1975) Structural Stability and Morphogenesis. W.A, Benjamin Inc, Reading, MA

[CR53] Tindall MJ, Porter SL, Maini PK et al (2008) Overview of mathematical approaches used to model bacterial chemotaxis II: bacterial populations. Bull Math Biol 70(6):1570–1607. 10.1007/s11538-008-9322-5 18642047 10.1007/s11538-008-9322-5

[CR54] Tindall MJ, Porter SL, Maini PK et al (2008) Overview of mathematical approaches used to model bacterial chemotaxis I: The single cell. Bull Math Biol 70(6):1525–1569. 10.1007/s11538-008-9321-6 18642048 10.1007/s11538-008-9321-6

[CR55] Vecchio DD, Qian Y, Murray R et al (2018) Future systems and control research in synthetic biology. Annu Rev Control 45:5–17

[CR56] Wang Y, Huang Z, Antoneli F et al (2021) The structure of infinitesimal homeostasis in input-output networks. J Math Biol 82:62. 10.1007/s00285-021-01614-1 34021398 10.1007/s00285-021-01614-1 PMC8139887

[CR57] Wyatt JK, Ritz-De Cecco A, Czeisler CA et al (1999) Circadian temperature and melatonin rhythms, sleep, and neurobehavioral function in humans living on a 20-h day. Amer J Physiol 277:1152–1163 10.1152/ajpregu.1999.277.4.r1152 10516257

[CR58] Xiao F, Doyle JC (2018) Robust perfect adaptation in biomolecular reaction networks. In: 2018 IEEE conference on decision and control (CDC), IEEE, pp 4345–4352

[CR59] Yi TM, Huang Y, Simon MI et al (2000) Robust perfect adaptation in bacterial chemotaxis through integral feedback control. Proc Natl Acad Sci 97(9):4649–4653 10781070 10.1073/pnas.97.9.4649 PMC18287

[CR60] Zeeman EC (1977) Catastrophe Theory - Selected Papers 1972–1977. Addison-Wesley, London

